# Effective Communication About Pregnancy, Birth, Lactation, Breastfeeding and Newborn Care: The Importance of Sexed Language

**DOI:** 10.3389/fgwh.2022.818856

**Published:** 2022-02-07

**Authors:** Karleen D. Gribble, Susan Bewley, Melissa C. Bartick, Roger Mathisen, Shawn Walker, Jenny Gamble, Nils J. Bergman, Arun Gupta, Jennifer J. Hocking, Hannah G. Dahlen

**Affiliations:** ^1^School of Nursing and Midwifery, Western Sydney University, Parramatta, NSW, Australia; ^2^Department of Women and Children's Health, King's College London, London, United Kingdom; ^3^Mount Auburn Hospital, Cambridge, MA, United States; ^4^Department of Medicine, Harvard Medical School, Boston, MA, United States; ^5^Alive & Thrive Southeast Asia, FHI Solutions, Hanoi, Vietnam; ^6^Chelsea and Westminster Hospital National Health Service (NHS) Foundation Trust, London, United Kingdom; ^7^School of Nursing and Midwifery, Griffith University, Brisbane, QLD, Australia; ^8^Centre for Health Care Research, Coventry University, Coventry, United Kingdom; ^9^Department of Women's and Children's Health, Karolinska Institutet, Stockholm, Sweden; ^10^Breastfeeding Promotion Network of India, New Delhi, India; ^11^School of Nursing, Midwifery and Paramedicine, Australian Catholic University, Melbourne, VIC, Australia

**Keywords:** breastfeeding, health communication, gender identity, inclusivity, mothers, pregnancy, sex, women

## Introduction

On 24 September 2021, The Lancet medical journal highlighted an article on its cover with a single sentence in large text; “*Historically, the anatomy and physiology of bodies with vaginas have been neglected.”* This statement, in which the word “women” was replaced with the phrase “bodies with vaginas,” is part of a trend to remove sexed terms such as “women” and “mothers” from discussions of female reproduction. The good and important intention behind these changes is sensitivity to, and acknowledgment of, the needs of people who are biologically female and yet do not consider themselves to be women because of their gender identity (1). However, these changes are often not deliberated regarding their impact on accuracy or potential for other unintended consequences. In this paper we present some background to this issue, describe various observed impacts, consider a number of potentially deleterious consequences, and suggest a way forward.

Sex (a reproductive category), gender (a societal role), and gender identity (an inner sense of self) are not synonymous (2, 3). Sex is salient to reproduction, as there are only two gametes and pubertal pathways to adulthood and gamete production, and only one gamete producing body type that becomes pregnant (2). As a general principle of communication it is well established that the sex of individuals should be made visible when it is relevant and should not be invoked when it is not (4–9). This facilitates avoidance of sex stereotyping while ensuring that sex-based needs and issues are not overlooked (4–9). In communication related to female reproduction, sexed language including the words “women” and “mothers” has therefore predominated. Yet, this usage has been challenged in response to rising numbers and visibility of people who have a gender identity which means they do not wish to be referred to as such (10, 11). As described below, we should address individuals as they wish (12), but more broadly there are risks to desexing language when describing female reproduction.

The discussion here is presented with an explicitly global audience in mind. While people who do not conform to the social expectations of their sex are ubiquitous throughout the world, the response to such individuals is influenced by culture in which they reside. This includes in the level of acceptance or marginalization they experience, the ways in which they are accommodated and the ways in which their non-conformity is conceptualized (13). It should be recognized that the penalty for non-conformity with gender roles can be high (14). Where the concept of gender identity is salient, desexing the language of female reproduction has emerged as an accommodation to remedy marginalization (10, 11). However, it needs to be kept in mind that pregnant and birthing women and new mothers and their infants have unique vulnerabilities and also require protection.

Each day, an estimated 810 women die during pregnancy, birth, and afterwards, with the majority of deaths in low- and middle-income countries (15). More women across low-, middle- and high-income countries suffer life threatening pregnancy and birth complications with short- and long-term consequences (16). Maltreatment and obstetric violence occurs everywhere and significantly contributes to birth trauma (17, 18). Puerperal psychosis affects 1–2 in every 1,000 mothers often in the first few days after birth (19) and is a leading cause of maternal death through suicide, as well as infanticide (20). Maternal deaths from non-medical causes such as suicide and injury, are gaining increased attention from leading health bodies such as the World Health Organization, with calls to extend reporting to a year after birth (21). Globally, 3.9 million infants die each year (22). Over 800 000 of these deaths are attributable to premature cessation of exclusive or any breastfeeding (23). Even in the wealthiest contexts, early discontinuation of breastfeeding is responsible for a large proportion of infant hospitalisations (24). Thus, Article 25 of the Universal Declaration of Human Rights says that the states of “*motherhood and childhood are entitled to special care and assistance”* (25) and the United Nations Convention on the Rights of the Child states that the best interests of the child are paramount (26).

## The Concept of Gender Identity

Gender identity can be described as an individual's internalized sense of being masculine, feminine or something else, or as an internal understanding of oneself as man, woman, both or neither, and is independent of sex (27, 28). The concept of gender identity originated in the 1960s in the United States of America (USA) (29), was refined in the 1990s through a postmodern philosophy called Queer Theory (30) and continues to evolve. Central to Queer Theory are the twin propositions that both sex and gender are socially constructed (31, 32) and that gender is the more important of the two (3, 33)^1^. Ideas informed by Queer Theory have spread from the USA to become influential in many other Western countries and beyond (30). The number of children, adolescents, and adults, reporting gender identities in conflict with their sex (described as being “transgender”) has grown dramatically in recent years (35). Alongside this increase, the idea that not everyone who gives birth is a woman has gained prominence.

Crucially, words such as “woman,” and “mother” can have *both* sexed *and* gendered meanings. The long-established sexed meanings are that “woman” means an adult of the female sex^2^, and “mother” means a female parent (36). However, when new Queer Theory-informed gendered meanings are applied, “woman” means an adult with a gender identity of “woman,” and “mother” means a parent with a gender identity of “woman” (37). The same principles apply to sexed and gendered understandings of “man” and “father.” In this paper, and the associated Supplementary Materials, unless otherwise stated, we use the sexed meanings of these words.

In response to Queer Theory-derived concerns, many organizations and individuals are changing the language they use to describe women so as to prioritize gendered understandings and avoid sexed terminology^3^ (38–41). These language changes are intended to avoid distress, are described as inclusive (42), and are encouraged by diversity, equity, and inclusion initiatives. Desexed language is most common in the English-speaking West, but it is increasingly being applied internationally (43–45). However, there appears to have been little consideration of the ethics of these changes, including the principles of avoiding harm and health maximization (46), or how they may impact on women and children's rights. The exercise of medical ethics requires balancing “autonomy” and “justice.” In this context, supporting autonomy is a legitimate and ethical goal, but the principles of distributive justice, to respect the equality, and dignity of every individual in the population must also be considered (47). Although the proposed language changes relate to women, they also impact children as the mother and infant form a dyad, whose physiologic functions depend on one another and are intimately interconnected in a unique, vital and transient developmental state (48–50). It behooves us therefore to be certain of how women's needs and children's developmental prerequisites may be affected by these changes in language and how they might impact advocacy for maternal and child health and human rights.

## How is Language Being Changed?

Avoidance of sexed terms most commonly results in the words “woman” and “women” being replaced with “person”, “people” or “families” and the words “mother” and “mothers” being replaced with “parent”, “parents”, “family” or “families” (51). Sometimes body parts (e.g. “vagina owners”) or processes (e.g. “birthers”) are also used. Terms such as “non-males” or “non-men” may be used to denote women. “Maternity” (52), “maternal” (53), “midwife” (54), and “breastfeeding” (52) have also become contentious terms.

Contrasting with the *either/or* of replacing sexed words, it is sometimes proposed to use *both/and* words. So, rather than referring to “women” or “mothers”, one might say “women and birthing people,” “women and other birthing people,” or “mothers and parents,” a strategy commonly described as “additive language” (54). Text may also be constructed so that “women,” “mothers” or an alternative to these terms are all avoided. Reference is made instead to “pregnancies,” “births,” or “infant feeding” / “breastfeeding”, circumventing entirely the need to indicate who experiences these states. The term “girls” may be avoided altogether with minors not disaggregated from adult women who are pregnant or mothers. Sometimes a mixture of sexed terms and some or all of these strategies may be used within the same document, described as “using a variety of terms” (55). Notably, desexing language in relation to males occurs less frequently (56). Some of the replacement desexed terms are shown in Table 1 and detailed examples of language changes and their interpretation can be found in Supplementary Material 1.

**Table 1:** Sexed terms and some of their replacement desexed terms.

**Sexed terms**	**Replacement desexed terms**
Birthing women	Birth givers, birth persons, birthers, birthing bodies, birthing families, birthing parents, birthing people, laborers
Breastfeeding	Body feeding, breastfeeding/chestfeeding, chestfeeding, feeding from the body, human milk feeding, lactating
Breastfeeding mothers	Breastfeeders, breastfeeding families, breastfeeding parents, chestfeeding parents, lactating families, lactating individuals, lactating parents, lactators
Breasts	Chest tissue, chests, glands, lactating tissue, mammary glands, mammary tissue
Breastmilk	Chest milk, human milk, parents' milk
Female	Woman (to mean anyone with the gender identity of woman irrespective of their sex)
Maternal	Parental
Maternity care	Perinatal care
Mother-infant dyad	Dyad, lactating dyad, parent-infant dyad
Mother-to-mother	Parent-to-parent, peer-to-peer
Mothers	Birthers, birthing parents, caregivers, families, gestational parents, parents, postnatal people, postpartum individuals
Non-pregnant women	Non-pregnant people, non-pregnant persons
Pregnant women	Birth givers, childbearing person, expectant parents, expectant persons, gestational carriers, gestators, pregnant families, pregnant patients, pregnant people, pregnant persons, service users
Sex	Gender, sex/gender
Women	Birth people, bodies with vaginas, cervix havers, menstruators, non-males, non-men, people, people with a cervix, people with vaginas, uterus havers, vulva owners

## What are the Consequences of These Language Changes?

Desexing the language of female reproduction has been done with a view to being sensitive to individual needs and as beneficial, kind, and inclusive. Yet, this kindness has delivered unintended consequences that have serious implications for women and children. These include: decreasing overall inclusivity; dehumanizing; including people who should be excluded; being imprecise, inaccurate or misleading; and disembodying and undermining breastfeeding. In addition, avoidance of the term “mother” in its sexed sense, risks reducing recognition and the right to protection of the mother-infant dyad.

### Decreases Overall Inclusivity

Avoiding sexed terminology in relation to female reproduction works against the plain language principle of health communication and risks reducing inclusivity for vulnerable groups by making communications more difficult to understand (57). Those who are young, with low literacy or education, with an intellectual disability, from conservative religious backgrounds, or being communicated to in their non-native language are at increased risk of misunderstanding desexed language (58–62). However, even women with high levels of education may not be familiar with female reproductive processes and terms of female anatomy and physiology and so may not understand some desexed terms (63–65). They may not know, for example, that “a person with a cervix” is a woman and refers to them (59). Translating desexed text into other languages may also be more difficult particularly when there is no direct equivalent to the English sex-neutral “parent” (e.g. Spanish which has only “padre”) (66).

### Dehumanizes

Numerous alternative terms for “women” and “mothers” involve references to body parts or physiological processes. Referring to individuals in this reduced, mechanistic way is commonly perceived as “othering” and dehumanizing (67). For example, the term “pregnant woman” identifies the subject as a person experiencing a physiological state, whereas “gestational carrier” or “birther” marginalizes their humanity. Efforts to eliminate dehumanizing language in medical care are longstanding (68), including in relation to women during pregnancy, birth, and new motherhood (67, 69–71). Using language that respects childbearing women is imperative given the prevalence of obstetric violence (18, 72, 73). Considering women in relation to males as “non-men” or “non-males”, treats the male body as standard (8) and hearkens back to the sexist Aristotelian conceptualization of women as failed men (74).

### Includes People Who Should Be Excluded

Terms such as “parents” and “families” as replacements for “mothers” can inappropriately include fathers and other family members, thus diminishing and invisibilising women (75). Use of “people” and “families” as replacements for “women” can similarly inappropriately include males and other family members. Women have unique experiences, needs and rights in relation to pregnancy, birth, and breastfeeding that are not shared with others (18, 76–79). It cannot be assumed that a woman's interests will align with those of her husband or partner. This is most clearly illustrated by the issue of domestic violence which often commences or increases during pregnancy and for which the worldwide prevalence ranges from 5 to 63% (80). Women, even when pregnant, do not lose their individual human rights, and should be supported to make autonomous decisions throughout pregnancy, birth, and breastfeeding. This includes for example, their companion of choice during birth (81) who may or may not be the father of their child. However, text referring to “birthing families” can suggest other family members have rights regarding a woman's decision making during birth. Similarly, text referring to supporting “parents” or “families” to make infant feeding decisions (82) suggests people other than the mother should make decisions regarding breastfeeding (75). This overlooks that partners and family members may directly or indirectly undermine breastfeeding (75, 83). It also obscures the positionality of women as rights-holders and family members as duty-bearers in relation to breastfeeding (76). Terminology that includes others can thus impede the provision of appropriate care and erode the rights of mothers and their infants.

The intent of “additive language” is to encapsulate pregnant and birthing females or female parents as a group but to do so in a way that avoids offense to those who do not wish to be named as women or mothers. However, the addition of terms like “birthing people” or “breastfeeding parents” changes the meanings of “women” and “mothers” from sexed terms that include all female people and all female parents, to gendered terms that may be confusing or inappropriately inclusive. For example, what does the phrase “women and birthing people” actually mean? This construction could be interpreted in a literal way as meaning that “women” are not people. Another interpretation occurs if “women” is meant or read in a gendered sense so including males with the gender identity of “woman” who cannot be pregnant or give birth. It is not always clear from the context. The change in meaning of “women” from a sexed term to a gender identity can also mean that those women who do not have a belief in gender identity as a concept do not see themselves reflected in the gendered use of “women.” Consequently, they may feel objectified by terms referring to processes like “birthing people” [e.g. (84)]. Thus, although sprinkling some “additive language” is often presented as a simple solution, it has its own risks, particularly when there is a need to be specific, to refer only to female people or female parents and to exclude male people or male parents.

### Introduces Inaccuracy, Precludes Precision, and Creates Confusion

Replacing a word with another of different meaning as if they are synonyms makes communications inaccurate or confusing. For example, in a growing number of papers, the severity of COVID-19 disease in pregnant women is being misrepresented by comparing “pregnant people” to “non-pregnant people” (40, 85–92) when the comparator in the research in question is “non-pregnant females.” Given the greater severity of COVID-19 disease in males (93), this misrepresentation means readers may under-estimate disease severity in pregnant women. Highly regarded organizations like the United States Centers for Disease Control and Prevention (40) and the Australian Department of Health (85) have made this error, and research containing this error has been published in the eminent New England Journal of Medicine (86). In the Australian Department of Health case, the mistake appeared when a previously published document was updated and a seemingly simple and innocuous “find and replace” undertaken with the word “women” switched with “people.” This change made the statistics on disease severity incorrect (see Supplementary Material 1 for further details). Carelessness may partly explain such errors, but there appears to be no easy way to straightforwardly communicate scientific information about female reproduction without using sexed terms. The misrepresentation of research and health communication during a pandemic ought to raise serious concern about how inappropriately desexing language can undermine public health.

Describing the frequency of sex-specific conditions referring to people rather than women as the denominator means incidence may be misreported. For example, it has been incorrectly stated that “1 in 8 *people”* develop breast cancer (94), that “8 in 10 *people”* will get pregnant after having unprotected sex (95), and that “1 in 10 *people”* have endometriosis (96) (our *emphases*). On the other hand, correctly stating that *1 in 20* people have endometriosis reduces the cognitive impact of the statistic because of the higher denominator and obfuscates a key feature of the condition: that sufferers are almost exclusively female, and males have virtually zero risk (with just a handful of cases ever described).

The term “chest feeding” can cause confusion because its meaning is often unclear. Some understand it to restrictively describe a situation where someone who has little or no breast tissue feeds a baby with infant formula or donor milk using tubing taped to their nipple. Others use it simply as another term for breastfeeding. In the midst of this confusion, the health consequences of not breastfeeding may be diminished if tube feeding infant formula is categorized as “breastfeeding/chestfeeding.” The word “breast” is a sex-neutral term which refers to the mammary glands of males and females. Referring to “chests” rather than “breasts” is medically inaccurate. The “chest” in medical terminology refers to the ribcage and everything within it and does not include mammary tissue (97). Chest pain may signify a serious heart or lung condition, whereas breast pain may signify a breast condition such as mastitis.

Desexed language can make it unclear who is being referred to. Does “breastfeeding people” mean mothers, infants or both? Are “postnatal people” those who have just given birth or those who are providing postnatal care? Using the phrase “breastfeeding parents” rather than “breastfeeding mothers” or “women,” both suggests the partner is participating in the act of breastfeeding and makes invisible the sex of the person breastfeeding the child. In this way, desexed language obscures the practical and power imbalances in relationships, decision making, and economics that breastfeeding mothers may face because they are female (98–102). Similarly, avoiding references to “girls” means that their very specific vulnerabilities as pregnant minors or minor mothers may be overlooked (103, 104). A mixture of sexed and desexed terminology within the same document can be particularly confusing [e.g. (105–107)]. Assisted reproductive technologies which can separate the genetic, gestational and social contributions to parenting increase the importance of accurate language rather than obscuring matters by using internally contradictory phrases such as “female sperm” as an example (108). We would argue that using “female” to describe a biologically male person with the gender identity of “woman” is inappropriate and that in order to accurately denote the sexes, “male” and female” should be retained as wholly sexed terms (109).

### Disembodies and Undermines Breastfeeding

Replacing “breastfeeding” with “human milk feeding” even when the mother is feeding directly from the breast, disembodies it and places emphasis on the milk as separate from the mother. The role of the breastfeeding mother becomes inconsequential and other individuals can be seen as equivalent caregivers even of very young infants. “Human milk feeding” places expressed milk feeding on an equal footing with breastfeeding thus supporting the trend toward predominantly, or exclusively, eschewing direct breastfeeding in favor of bottle feeding expressed milk that has been noted in some countries (110–112). Expressed milk feeding has significant drawbacks from a public health perspective, including reduced duration of infant access to breastmilk (113, 114) and poorer infant health outcomes (115, 116) and so should not be encouraged over direct breastfeeding. The disembodiment of breastfeeding makes breastmilk just another type of milk and so elides with market interests, relating to selling breastmilk substitutes (including commercial infant formulas), breast pumps, and feeding bottles. It also works against efforts to recognize the unique relational aspects of breastfeeding that support maternal caregiving capacity and infant mental health (50, 117).

### The Significance of the Word Mother

There is a word for mother in every language. It is commonly the first word said by children and is perhaps the oldest word ever spoken (118). “Mother” holds meaning beyond that of “female parent” containing connotations of “nurturing”, “nourishing,” “love”, “responsibility”, and “child rearing” that support the importance of mothers to children (119–121). When undermining the mother-infant relationship is desired, mothers may be described differently. For example, women whose children have been adopted by someone else are often called “birth mothers” (122–124). It has been noted that the addition of “birth” modifies the association between “mother” and “rearing,” “nurturing,” and “responsibility” suggesting that the relationship is limited to the act of birthing (121, 125, 126). The term “birth mother” is also used for pregnant women who are being encouraged to place their infant for adoption and so be separated, as well as for women whose children have been removed from their care because of maltreatment (123, 127). “Birth mother” thus minimizes and marginalizes (123, 124). Similarly, in surrogacy arrangements, a desire to avoid any of the relationship connotations of “mother” means that the word is avoided altogether. Such women may be referred to as “surrogates,” “gestational carriers,” or even as “a surrogate uterus” (128, 129). Even though some women may apply these terms to themselves in order to distance psychologically from the children they bear, the words act to dismember and objectify them and reduce their importance to the infant.

The alternative desexed terms for “mother” have similarities to these modifications by consisting first of avoiding “mother” and/or the subsequent addition of an adjective referring to a process (birth, lactation, or breastfeeding). These language changes have the potential, through linguistic processes, to undermine recognition of what mothers mean to all infants. This undermining may have the most deleterious impact in situations of adversity where mothers and infants are most in need of protection: such as circumstances of high maternal or infant mortality; during disasters; when infants are sick or premature; or where the mother-infant relationship is in jeopardy due to poverty, intergenerational trauma, domestic violence, incarceration, poor maternal mental health, or substance use. Desexed language can make it particularly difficult to identify mothers who are not breastfeeding, even though they should be considered a vulnerable group (130). It is noteworthy that the term “Kangaroo Mother Care” was created to emphasize the unique importance of mothers to their sick and premature infants. The chest wall and breasts of a lactating mother are warmer than those of a non-lactating woman or man (131). This is a highly conserved neuroendocrine physiological expression of primate reproductive fitness for all newborns, and more so for infants born small or sick (132). However, some are now suggesting that the name be changed to “Kangaroo Parent Care” in the pursuit of inclusivity and a misguided attempt at equality (133). This has already been implemented in some hospitals (134).

### Broader Issues for Policy and Advocacy

Imprecise or inappropriate terminology can have particular implications in policy which may affect many women and infants. For example, during the COVID-19 pandemic, Neonatal Intensive Care Unit policies commonly restricted visiting to a single person for a limited period per day (135). Visiting policies that referred to “parents”, and did not differentiate between fathers and mothers or prioritize mothers, resulted in increased mother-infant separation with an associated adverse impact on breastfeeding and the mother-infant relationship (135, 136).

It has long been recognized that language plays a role in advocacy. For this reason, feminist linguistic activism of the 1970-80s sought to remove sexist language from public life (137). Women used to be invisible when “he” or “men” were used as the default contributing to the disregard of women in research, policy, and public life (138). In the midst of the current move to desex language, we argue that if women and mothers are not named, it makes it more difficult to effectively advocate for them; “women” disappear into “people” and “mothers” disappear into “parents.” This inevitably changes the focus. For example, the World Alliance for Breastfeeding Action (WABA) recently moved away from matricentric language and began campaigning for “*the integration of parents' reproductive work”* in the workplace (45). This shift is risky because, while some countries provide in excess of 12 months paid maternity leave alongside leave for fathers/partners (an ideal situation), other countries have no, or relatively short, maternity leave (139). Nonetheless, in some countries with short maternity leave provision, “parental leave” lobbying is focused on providing paternity leave that would not be available to mothers rather than on maternity leave extension (140, 141). In this context, WABA's shift to advocate for workplace leave for “parents” rather than just “mothers” diminishes advocacy for the leave necessary to support the breastfeeding rights of women and children (76, 130).

### Broader Issues for Research

Use of precise language and definitions in communicating and designing research regarding female reproduction and newborn care is vital. New “parents” do not have the same health needs or experiences as new “mothers” although language in publications or research design that does not distinguish between these groups can suggest that they do [e.g. (136)]. Research on women's health or maternal and child health that is de-sexed may be more difficult to find as search terms disappear and so result in it being overlooked in indexing for initiatives such as the United Nations Sustainable Development Goals (142). Already, journal styles may be preventing authors from using “women” or “mothers” in a sexed sense (39). Advocacy to reduce the female data gap in medical and other research is ongoing (138, 143, 144). Yet, it is being suggested that collection of data on gender identity should be prioritized over sex (145) or that data on sex should not be collected at all (33). There have been reports of ethics committees rejecting research applications that include a sex question (146). There is also confusion around what is meant by sex and gender, and as previously noted, gender is sometimes used as a synonym for sex (3). This use of “gender” when meaning “sex,” while common, including by United Nations organizations, is contributing to confusion on what data is being collected or represented and more generally who is being advocated for. We argue that if sex is meant, sex should be the term used. Clarity about definitions, and the separate collection of data on sex and gender identity (where relevant) is needed (27). As there are very few areas of life that are not impacted by sex, we postulate that there are very few areas of human research where data on sex should not be collected (3). To do otherwise risks undermining efforts to reduce the female data gap (138) to the detriment of women and children.

## Cultural Imperialism in Global Public Health

As previously explained, the impetus to desex language in relation to female reproduction flows from a philosophy developed in the USA and within which American understandings and priorities predominate (147). In the context of global public health, an increasing encouragement, or requirement, to desex language by international organizations or funders based in the USA/the West may be experienced not only as confusing but also as cultural and linguistic imperialism (148, 149). This view was recently expressed by over 250 breastfeeding counselors from 45 countries to the Board of the USA-based breastfeeding support organization La Leche League who stated that changes in language requirements were being experienced as “colonialist” and “oppressive” (150). Organizations and individuals have a responsibility to avoid imposition of Western ideas that may cause harm to those with whom they work^4^. In addition, the risks associated with desexing language should be carefully considered with an impact assessment undertaken, even where the concept of gender identity holds cultural salience.

## Ways Forward for Those Who are Pregnant, Birthing, and Breastfeeding But Who Do Not Identify as Women or Mothers

In some circumstances, application of gendered rather than sexed meanings of words is appropriate and we fully endorse the importance of being inclusive and respectful (12). For those who are pregnant, birthing, and breastfeeding but who do not identify as women, the individual's preferred terminology for themselves and their body parts should be used wherever possible (1, 151). This may mean avoiding sexed language altogether and using gendered terms on this one-to-one basis. However, preferred language usage should not be presumed for anyone (151). Targeted public health campaigns should address the needs of this group, not just to ensure that the language used is suitable (152) but because they may have additional social needs or medical needs associated with treatments such as testosterone use or surgery to remove breast tissue (153, 154). Similarly, targeted health support materials are of value and have already been produced by some organizations [e.g. (155–157)]. Development of separate desexed materials (much as might occur for people from different language backgrounds) may be a useful strategy [e.g. (158, 159)].

The inclusion of all who are pregnant, birthing or breastfeeding, independent of their gender identity, can be made explicit through inclusivity statements or definitions, some examples of which are provided in Supplementary Material 2. These adaptions may be suitable in circumstances where desexing language is not appropriate because of the negative impact on clarity or any of the other above-mentioned detriments. For example, the United Kingdom NICE Postnatal Care Guideline states that, “*The guideline uses the terms “woman” or “mother” throughout. These should be taken to include people who do not identify as women but are pregnant or have given birth”* (160). A variety of strategies may be appropriate in different circumstances. We recognize that it is a continuing challenge to apply language in a way that is clear, concise and preserves the dignity of all people being described.

There are concerns related to medical records of transgender people. In some countries, individuals are able to change sex markers in their medical records on the basis of their gender identity (109, 161). The case of an inaccurate sex marker which may have contributed to a delayed identification of pregnancy with a resultant stillbirth, illustrates the potential adverse impact of this practice (162). Problems associated with cervical cancer screening have also been reported where sex markers have been altered (163). The Gender Dysphoria Alliance of Canada, have emphasized the importance of sex being accurately recorded in health records and for the need for clear language around sex (164). Noting both sex and gender identity (where relevant) in medical records may provide a solution (2, 165). It needs also to be stressed that that there is a dearth of knowledge about how to support the health needs of transgender individuals, particularly regarding the long-term health effects of hormonal treatments. Further research is urgently needed and in this research, data on sex remains vital. Those who do not identify as women or mothers, and yet become pregnant and give birth, are most benefited by a culture and health service that recognizes and deeply understands the underlying molecular, cellular, behavioral and organismal aspects required to reproduce. Included in such understandings are adaptation and possible accompanying allostatic load with maladaptation when the biological expectation is not met (166–168). Such maladaptation can be averted and managed if it is properly characterized from the basic biology and use of clear terminology (169).

## Recommendations and Conclusion

The purpose of this paper is to increase understanding of why and how language regarding pregnancy, birth, lactation, breastfeeding, and newborn care is being desexed and to empower readers to consider the implications of these changes in their own contexts. The issues raised here are relevant to other situations where sex is central, including: domestic violence, sexual assault, sex-selective abortion and infanticide, female genital mutilation, and reproductive cancers and other sex-specific conditions. Different approaches to describing women and mothers may be applicable in different contexts and with different purposes. Regardless of the approach taken, we would recommend that clarity in terminology and avoiding conflation of terms are important. In particular, when sex is meant, refer to “sex”; when gendered expectations of the sexes is meant, use “gender” and clearly define the term; and when “gender identity” is meant, ensure the phrase is distinguished from both gendered expectations of the sexes and from sex itself. We suggest consideration of the following questions: How can I be clear? How can I include the people who should be included and exclude the people who should be excluded? How can I ensure that people understand what I mean and can readily recognize themselves? How can I avoid dehumanizing language? Does it make sense to apply a gendered understanding of words or a sexed understanding? Am I engaging in cultural imperialism or improper use of privilege by requiring others to use language in a particular way? How does language usage support or undermine the rights of women and children? When should I make compromises regarding clarity or precision or any other detriments to desexing language and how can I mitigate against these detriments? How can I ensure that the special needs of those who are female, but who have a gender identity they experience in conflict with their sex, are met? Some have argued that objections to desexing the language of female reproduction can only be rooted in prejudice or resistance to change (170). However, as we have described, there are significant implications to desexing language when referring to inherently sexed processes and states. These implications need to be openly discussed and thoughtfully considered. We hope that this paper assists in such discussions.

## Author Contributions

KG conceived of and wrote the first draft of the paper. KG, SB, MB, RM, SW, JG, NB, AG, JH, and HD discussed and provided original content for the paper. All authors critically revised and approved the manuscript.

## Conflict of Interest

The authors declare that the research was conducted in the absence of any commercial or financial relationships that could be construed as a potential conflict of interest.

## Publisher's Note

All claims expressed in this article are solely those of the authors and do not necessarily represent those of their affiliated organizations, or those of the publisher, the editors and the reviewers. Any product that may be evaluated in this article, or claim that may be made by its manufacturer, is not guaranteed or endorsed by the publisher.

## Footnotes

^1^Another idea associated with Queer Theory is that sex is a spectrum or that there are more than two sexes. Individuals with intersex variations may be brought in as proof of this. However, the conceptualisation of people with intersex variations as being of a “third” sex is misconstrued and has been opposed by intersex organizations (34).

^2^In this sexed meaning, everyone who gives birth is indeed a woman.

^3^Sometimes the desexing of language is referred to as using “gender neutral” or “non-gendered” language. Such characterisation is misleading. Gender neutral or non-gendered language more properly means not ascribing a gender role when the sex of the person undertaking the role is irrelevant. For example, as they can be male or female is it appropriate to avoid “police*men*” and instead refer to “police *officers*”.

^4^This includes understanding that an interpretation of gender non-conformity in non-Western cultures through a Queer Theory lens risks misunderstanding and neocolonialism, see (13).
