# Supplementary material for: Effective Communication About Pregnancy, Birth, Lactation, Breastfeeding and Newborn Care: The Importance of Sexed Language
Source: Front Glob Womens Health. 2022 Feb 7;3:818856. doi: 10.3389/fgwh.2022.818856 (PMC8864964; doi:10.3389/fgwh.2022.818856)
Supplement: Supplementary file 1 [file Table_1.doc]

**Supplementary Material 1- Example uses of desexed language with explanation of implications (sexed language is used in all explanations unless otherwise indicated)**

| **Quotation** | **Explanation** |
| --- | --- |
| **Misrepresentation of research by replacing *women* with *people* or *mothers* with *parents* or *families*** | |
| *COVID-19 vaccination decision guide for* ***women*** ***who are pregnant****, breastfeeding or planning pregnancy*  Version 4.1 (17/6/21)  ***Pregnant women*** *with COVID-19 have a higher risk of certain complications compared to* ***non-pregnant women*** *with COVID-19 of the same age, including: An increased risk (about 5 times higher) of needing admission to hospital. An increased risk (about 2-3 times higher) of needing admission to an intensive care unit. An increased risk (about 3 times higher) of needing invasive ventilation (breathing life support).*  *COVID-19 vaccination decision guide for* ***people who are pregnant****, breastfeeding or planning pregnancy*  Version 5.1 (19/8/21)  ***Pregnant people*** *with COVID-19 have a higher risk of certain complications compared to* ***non-pregnant people*** *with COVID-19 of the same age, including: an increased risk (about 5 times higher) of needing admission to hospital; an increased risk (about 2-3 times higher) of needing admission to an intensive care unit; an increased risk (about 3 times higher) of needing invasive ventilation (breathing life support).*  *COVID-19 vaccination decision guide for* ***women******who are pregnant****, breastfeeding or planning pregnancy*  Version 6 (2/9/21)  ***Those who contract COVID-19 whilst pregnant*** *have a higher risk of certain complications compared to* ***those who are not pregnant with COVID-19*** *of the same age, including: • An increased risk (about 5 times higher) of needing admission to hospital. • An increased risk (about 2-3 times higher) of needing admission to an intensive care unit.• An increased risk (about 3 times higher) of needing invasive ventilation (breathing life support).*  *COVID-19 vaccination decision guide for* ***women who are pregnant****, breastfeeding or planning pregnancy*  Version 6 (15/9/21)  ***Women who contract COVID-19 whilst pregnant*** *have a higher risk of certain complications compared to* ***non-pregnant women*** *of the same age who contract COVID-19, including: • An increased risk (about 5 times higher) of needing admission to hospital.• An increased risk (about 2-3 times higher) of needing admission to an intensive care unit. • An increased risk (about 3 times higher) of needing invasive ventilation (breathing life support).* | Australian Government Department of Health  Early versions of this vaccination guide for pregnant and breastfeeding women contained only sexed language. For Version 5.1 a simple ‘find and replace’ *women* with *people* was undertaken resulting in some content incorrectly representing the research on COVID-19 severity in breastfeeding women. Version 6 of the guide used a mix of sexed and desexed language but some of the desexed language continued to misrepresent research outcomes. A second version of Version 6 was published wherein these errors were corrected and there was no misleading use of desexed language.  Parts of the desexed versions of this decision guide appear to have been copied by the Ministry of Health in South Australia with the errors replicated (see next example).  <https://www.health.gov.au/resources/publications/covid-19-vaccination-shared-decision-making-guide-for-women-who-are-pregnant-breastfeeding-or-planning-pregnancy> |
| *Frequently Asked Questions: COVID-19 Vaccination and Pregnant, Planning a Family, and Breastfeeding*  *Those who are* ***pregnant*** *and their unborn baby have a significantly higher risk of severe outcomes from COVID-19 than* ***non-pregnant people:***  *> 5 times higher risk of requiring a hospital admission.*  *> 2-3 times higher risk of needing treatment in a hospital intensive care unit.*  *> 1½ times higher chance of being born preterm or admission to a special care nursery*. | South Australia Health  This document uses *women* in a sexed way throughout except for a section that appears to have been copied from the Australian Department of Health *Shared* *Decision Making Guide for Women who are Pregnant, Breastfeeding or Planning Pregnancy*. It copies the error that was created in that document when it was desexed, and while the source document has been corrected, the South Australia Health document has not. This provides an example of how errors introduced by desexing language can be spread.  <https://www.sahealth.sa.gov.au/wps/wcm/connect/public+content/sa+health+internet/conditions/infectious+diseases/covid-19/vaccine/covid-19+pregnancy+planning+a+family+and+breastfeeding/covid-19+vaccination+and+pregnancy+planning+a+family+and+breastfeeding> |
| Shimabukuro, T. T., Kim, S. Y., Myers, T. R., et al. (2021). Preliminary Findings of mRNA Covid-19 Vaccine Safety in Pregnant Persons. *New England Journal of Medicine, 384*(24), 2273-2282.  ***Pregnant persons*** *with Covid-19 are at increased risk for severe illness (e.g., resulting in admission to an intensive care unit, extracorporeal membrane oxygenation, or mechanical ventilation) and death, as compared with* ***nonpregnant persons*** *of reproductive age.* | This research compares *pregnant persons* to *non-pregnant persons* when the true comparator in the research cited is non-pregnant women. Given that males experience more severe COVID-19 disease than females, this mistake may result in an underestimation of the risks COVID-19 poses to pregnant women.  [*https://www.nejm.org/doi/full/10.1056/nejmoa2104983*](https://www.nejm.org/doi/full/10.1056/nejmoa2104983) |
| Twitter@GOLDMidwifery  @GOLDMidwifery: *Hyper- & hypo- arousal during pregnancy can be common* ***for families that have experienced previous losses***  @ProfJennyGamble: *Common in* ***mothers****. I am not sure there is data indicating it is common in* other family members  @RuthOshikanlu: ***I did say in mothers****, my focus was on the women in our care. It was misquoted. You are right. Thanks for correcting what was said.* | GOLD Midwifery  A conference representative misrepresented the content of a presentation on Twitter by referring to the experiences of *families* rather than *mothers.* This error was subsequently corrected by the presenter.  <https://archive.vn/lv1nj> |
| **Inappropriate inclusion** | |
| ANU Gender Institute, & ANU Centre for Learning and Teaching. (2021). Gender-Inclusive Handbook. Canberra: Australian National University.  *A number of studies indicate that the* ***parent’s capacity to feed their baby with human milk is impacted by the return to work or study.*** | Australian National University  This handbook states that return to work impacts the *parent’s* ability to feed their baby human milk, with the use of desexed language suggesting that this applies to both mothers and fathers. However, it is only the mother’s return to work that impacts the ability of infants to be fed breastmilk. |
| *Support research into donated breastmilk to help premature babies*  *We have an exciting opportunity to look at the broader impacts beyond infant health too, like whether having donor milk available* ***supports parents to reach their breastfeeding goals*** *and feel more empowered when caring for their newest addition.* | Australians Investing in Women  This website’s use of the word *parents* connected to *breastfeeding goals* suggests that fathers might have goals in relation to breastfeeding that should be supported when it is only mothers who should have goals and decision-making supported in relation to breastfeeding.  [https://web.archive.org/web/20210309014723/https://www.aiiw.org.au/projects-post/support-research-into-donated-breast-milk-to-help-premature-babies/](https://web.archive.org/web/20210309014723/https:/www.aiiw.org.au/projects-post/support-research-into-donated-breast-milk-to-help-premature-babies/) |
| *Advocating for Evidence-based Care*  *After that experience, she decided to become more involved in examining the evidence on breech positioning and its different delivery options, as well as advocating for* ***pregnant families’ rights****.* | Evidence-based birth podcast  This podcast’s use of *pregnant families* in relation to *rights* erroneously suggests that people other than the pregnant woman have rights in health care during pregnancy and birth.  <https://evidencebasedbirth.com/advocating-for-evidence-based-care-with-carissa-hipsher/> |
| *Informed consent in pregnancy and birth*  ***Informed consent****: that is a buzz-phrase for many* ***pregnant families*** *as they get ready to give birth…Informed consent starts with taking the time to educate yourself, asking questions, and making sure that you feel comfortable with the way that information is shared and decisions made in your pregnancy and birth.* | Tender Beginnings Birth and Parenting Services  This business’ use of *pregnant families* connected to *informed consent* suggests that people other than the pregnant woman are able to consent to health care provision in pregnancy and birth.  <http://atenderbeginning.com/blog/2015/9/8/informed-consent> |
| Twitter@lalecheleague  *The International Code of Marketing of Breastmilk Substitutes is of critical importance to the* ***health and well-being of babies and parents*** *globally.* | La Leche League International (LLLI)  LLLI’s use of *parents* erroneously suggests that the health of fathers is impacted when breastfeeding is impeded by the unethical marketing of breastmilk substitutes, however, it is only mothers’ health that is so impacted.  <https://twitter.com/lalecheleague/status/1380183771752849411> |
| *Empowering Parents Campaign*  *The Empowering Parents Campaign promotes social protection that will* ***facilitate the integration of parents’ productive and reproductive work*** *in both formal and informal settings*. | World Alliance for Breastfeeding Action (WABA)  WABA refers here to *parents’* *reproductive work* in the workplace. Women undertake reproductive work in the workplace during pregnancy and breastfeeding. Maternity workplace protections, including maternity leave and lactation breaks, enable women’s participation in the workforce. However, use of *parents* suggests that the reproductive work of males should also be supported in the workplace. It is difficult to imagine what this work would involve and how this might support breastfeeding.  <https://waba.org.my/epc/> |
| *Twitter@WHO*  *When* ***parents*** *are allowed to stay with their newborns, it* ***promotes early & effective breastfeeding****, including for low birthweight and #preterm babies. Separating* ***parents*** *&* ***babies*** *immediately after childbirth should be discouraged. #WorldPrematurityDay* | World Health Organization (WHO)  This tweet from the WHO erroneously states that keeping *parents* and *infants* together promotes breastfeeding when it is only keeping *mothers* and *infants* together that enables breastfeeding. There is no evidence that the presence of fathers increases breastfeeding in premature infants.  <https://twitter.com/WHO/status/1461228730521694209> |
| *Cervical cancer overview*  ***If you have a cervix*** *and have had any kind of sexual contact, you could get cervical cancer.*  *Cervical cancer mostly affects* ***people*** ***aged 30 to 50*** *who have ever been sexually active.*  *Every year in Ireland about* ***300 people*** *get cervical cancer.* ***90 women*** *die from it.*  *In* ***women aged 25 to 39*** *years, cervical cancer is the second most common cause of death due to cancer.*  *Cervical cancer is very rare in* ***people*** *under 25.* | Health Service Executive Ireland (HSE)  This webpage from HSE on cervical cancer uses a mix of sexed and desexed language alongside medical terminology making the text confusing. Individuals are told that if they *have a cervix* they could get cervical cancer and then told that it mostly affects *people* *30-50 years* and that *300 people* get cervical cancer each and *90 women* die from it. Readers may not know that it is women and only women who have a cervix and be confused that 300 people get cervical cancer and 90 women die and wonder why the treatment for cervical cancer for men is so much better than for women.  <https://www2.hse.ie/conditions/cervical-cancer/overview/> |
| Twitter@ILCA1985  *When* ***families*** *can’t reach their infant feeding goals, it affects communities. That’s why we are working to* ***make sure that the needs of breastfeeding and chestfeeding families*** *- and of skilled lactation providers- are heard on the world stage.* | International Lactation Consultant Association (ILCA)  ILCA’s use of *families* in relation to *infant feeding goals* suggests that in addition to mothers, fathers and other family members can and should have infant feeding goals. There is no awareness reflected that there may be a conflict between the wishes of the woman and her partner or her mother-in-law or mother.  <https://twitter.com/ILCA1985/status/1237108316188352513> |
| Twitter@USbreastfeeding  ***Breastfeeding families*** *throughout the United States are facing barriers that make it difficult or impossible to start or continue breastfeeding – but it does not have to be this way.* | United States Breastfeeding Committee (USBC)  USBC’s use of *families* suggests that individuals beyond the mother breastfeed and face difficulties undertaking breastfeeding when it is only mothers who do so. This usage also ignores that partners and family members are commonly the source of barriers that make breastfeeding difficult.  <https://twitter.com/USbreastfeeding/status/1372567277581778954> |
| Twitter@PPFA  Cervical cancer can **impact anyone** but some are more at risk than others. We are proud to provide care no matter what so that everyone can have access to preventative health services. | Planned Parenthood  By stating that cervical cancer can *impact anyone*, Planned Parenthood erroneously suggests that males can also develop this type of cancer and need screening. Such misinformation may undermine health communication regarding this disease as well as women’s understanding of their anatomy.  https://twitter.com/PPFA/status/1478773285987004420 |
| Spatz, D. L. (2020). Using Gender-Neutral Terms in Lactation. *MCN- American Journal of Maternal and Child Nursing, 45*(1), 61.  *At Children’s Hospital of Philadelphia, our hospital-wide breastfeeding committee recently reviewed and approved our updated milk expression log for* ***our families****. For example, the log book used to be called* ***“Mom’s Pumping Log”*** *and now it is called* ***“Family’s Pumping Log****: A Resource for Pumping Human Milk.”* | Children’s Hospital Philadelphia  The decision of the hospital to change from describing milk expression records from *Mom’s Pumping Log* to *Family’s Pumping Log* obscures that it is mothers who engages in the hard work of expressing milk for premature infants in Neonatal Intensive Care Units and who are in need of support and recognition.  [*https://pubmed.ncbi.nlm.nih.gov/31815798/*](https://pubmed.ncbi.nlm.nih.gov/31815798/) |
| Twitter@USbreastfeeding  *Continuity of Care in lactation support is about warm and coordinated hand-offs and transitions. Then, the needs of* ***lactating families*** *and those who support them are anticipated and met, #EveryStepOfTheWay.* | United States Breastfeeding Committee (USBC)  USBC’s use of *lactating families* suggests that the physiological state of lactation is being experienced by multiple family members instead of just mothers.  <https://twitter.com/USbreastfeeding/status/1421630510258954241> |
| *LLL USA Statement in Support of* ***Pumping Families***  *LLL USA supports all* ***families who exclusively express or pump*** *for their nurslings, no matter their reasons. As we often say in meetings, you are the expert on your family.* | La Leche League USA (LLLUSA)  LLLUSA’s reference to *families* who exclusively express milk ignores that it is women who are the ones who engage in the time consuming and difficult work of exclusively expressing breastmilk for their babies and who are in need of support.  <https://lllusa.org/wp-content/uploads/2019/10/2019-10-4-LLL-USA-Statement-in-Support-of-Pumping-Families.pdf> |
| **Additive language confusion** | |
| *New study suggests women and pregnant people are underrepresented in research to inform nutrition reference values* ***Women and pregnant people*** *are underrepresented in the studies used to draw up the nutritional guidance provided to Americans and Canadians, according to a paper published today in* Science Advances*…The team found that nearly one quarter of the studies included* ***men*** *only, and in research that did include* ***women, female participants*** *were underrepresented compared to* ***men****.* | Eureka alert  In this article, the phrase *women and pregnant people* suggests that a gendered understanding rather than a sexed understanding of *women* is being applied as *women* and *pregnant people* appear to be different groups of people. If a gendered understanding is read this means that males with a gender identity of woman might be included in the term *women*. However, a reading of the whole article makes it appear that what the article is describing is research showing that female people (i.e. women in the sexed sense) as a group, including those who are pregnant, are underrepresented in nutrition research. So in reality the concern is regarding the underrepresentation of those of the female sex as a whole, including the subset of this same group who are pregnant. The linguistic separation of women into two separate groups is thus, artificial and confusing.  <https://www.eurekalert.org/news-releases/932092> |
| *Toward Equitable Treatments for Women’s Health During Coronavirus and Beyond* *As the United States and the rest of the world rush to develop an effective vaccine and treatments for COVID-19, it is critical that these therapies work for everyone. Unfortunately, clinical trials and research have historically prioritized* ***white, cisgender men****.* ***Women****,* ***along with transgender men and nonbinary and gender-nonconforming people****, have been excluded and underrepresented—with consequences for their health. No measures to treat and prevent the coronavirus will be truly successful if they are not effective for large swaths of the population; a failure to provide a vaccine and treatment that works for* ***women*** *is a failure to protect the public’s health more broadly. Therefore, to better understand and address the different ways in which COVID-19 and other health conditions manifest—including but not limited to common conditions that disproportionately affect* ***women****, such as lupus and fibroids—it is critical for research and data to include* ***women****—especially* ***women of color and pregnant women—as well as transgender men and nonbinary and gender-nonconforming people****…*It is important to note that the study, like many studies, evaluated the inclusion of patients in a **male-female binary**; sex-disaggregated data often exclude **nonbinary and gender-nonconforming people** as well as exclude or obscure data for **transgender people**, who may not be categorized according to their gender identity if they are included at all. The **male bias** in clinical studies extended to research on animals as well: In a study of biomedical research on mammals, **male bias** was present in 8 out of 10 biological fields. | Center for American Progress  The goal of this web article appears to be to inform readers about the female data gap in medical research. However, the text confusingly says that there needs to be more research including *women* in a seemingly gendered sense of the word and then adds as well as *transgender men* (who are female) as well as *non-binary and gender-nonconforming people* who could be individuals of either sex. That the article also includes clearly sexed terms like *male* and *female* and criticises sex disaggregation as opposed to gender identity disaggregation of research adds further to the confusion and undermines advocacy to narrow the female data gap.  <https://www.americanprogress.org/issues/women/reports/2020/07/15/487429/toward-equitable-treatments-womens-health-coronavirus-beyond/> |
| *Unlocking the Mysteries of Human Lactation: Lactation Among Women and Parents Living with HIV- Evidence for a Change in US Policy* *Please join us for a special 2 hour learning experience on HIV and* ***lactation*** *in the USA. Although the current US Centers for Disease Control recommends against* ***breastfeeding*** *while living with HIV, there is growing evidence that there are circumstances when it is safe for* ***parents*** *living with HIV to* ***breastfeed or chestfeed****. We will share stories and experiences of* ***lactating parents*** *in the USA living with HIV, and the challenges they face within their medical communities. We will also describe documented inequity of care, including criminalization, that* ***lactating parents*** *living with HIV face based on race/ethnicity. We will wrap up with recent research evidence that demonstrates safety of* ***direct infant feeding*** *for* ***parents*** *living with HIV, how this could influence US public policy, and means of support that physicians can provide for* ***lactating parents*** *living with HIV.* | Institute for the Advancement of Breastfeeding Lactation and Education  This summary of a presentation on HIV and breastfeeding in the USA uses the terms *women*, *parents*, *lactation, lactating,* *breastfeeding*, *chestfeeding* and *direct infant feeding*. This mix of terms is confusing. Reference to *parents chestfeeding* allows for a reasonable interpretation that this is something that fathers might do with supplemental infant formula being provided at their chests using a supply line. This interpretation is supported by the use of the term *direct infant feeding* as the term *infant feeding* is often used to encompass both breastfeeding and formula feeding. The suggestion that some sort of mixed breastfeeding/formula feeding by HIV positive *parents* might be appropriate is concerning given the high risk of HIV transmission that occurs in mixed feeding situations.  <https://lacted.org/product/research202111/?fbclid=IwAR3D8_AWSom-tj4xYbKhgnO-Mjlyb_JMkeNGvcRu3E3_kXrY-RsdoxKw1Lo> |
| **Incorrect denominators** | |
| Facebook@Qendo  *March is Endometriosis Awareness Month, and at QENDO we’re sharing stories from the* ***1 in 10 people*** *affected by endometriosis in our community.* | QENDO  QENDO is a support organisation for *anyone affected by endometriosis, adenomyosis, PCOS, infertility or pelvic pain*. While their focus is on female reproductive health, their website and social media is desexed. When presenting prevalence of endometriosis as affecting 1 in 10 women, QENDO did not adjust the statistic during desexing for a mixed sex population and so erroneously stated that 1 in 10 *people* are affected by endometriosis rather than 1 in 20 *people*.  <https://www.facebook.com/qendo/posts/10158060320838596> |
| *1 in 10: Endometriosis Impacts College Students* *Even though endometriosis isn’t often talked about,* ***1 in 10 people*** *are diagnosed with it, which is about the* ***same number of people who are diagnosed with diabetes****.* | College Avenue Magazine This article for university students incorrectly states that 1 in 10 *people* will be diagnosed with endometriosis rather than 1 in 10 *women*. It is clear that when the text was desexed to present prevalence using a mixed sex population, the statistic was not adjusted to correctly represent endometriosis as affecting 1 in 20 *people*. The text is further confused by a comparison being made to diabetes prevalence, which is not impacted by sex. Thus, it is stated that diabetes and endometriosis have similar prevalence when overall, diabetes is twice as common as endometriosis across the population, although of similar prevalence in women.  <https://collegeavemag.com/endometriosisimpactscollegestudents/> |
| Trying to get pregnant  ***8 in 10 people under 40 will get pregnan****t within 1 year of trying by having regular sex without contraception* | UK National Health Service  In this web article from the NHS, the desexing of information for women about the process of conception results in a statement suggesting that either sex can get pregnant.  <https://web.archive.org/web/20201229204603/https://www.nhs.uk/pregnancy/trying-for-a-baby/trying-to-get-pregnant> |
| Rymaszewski, A. (2019). Interviewing breast cancer patients to understand the patients’ relationship with food before and after cancer diagnosis.  *In 2017, it is estimated that* ***318,590 Americans, men and women****, will be diagnosed with breast cancer. It was predicted in the 1970s that* ***1 in 11 Americans*** *would be diagnosed with breast cancer, currently, it is* ***1 in 8 people****.* | Clinicaltrials.gov  This registered trial description states that 1 in 8 *people* in the USA will be diagnosed with breast cancer. However, it is 1 in 8 *women* who will experience breast cancer as compared to only 1 in >800 *men*. This means that the correct statistic is that 1 in 63 *people* will be diagnosed with breast cancer.  https://clinicaltrials.gov/ct2/show/record/NCT04117438. |
| *How Joni is drawing awareness to menstrual issues in a stigmatized industry* *According to a 2018 report by Plan International Canada,* ***one in three Canadians*** *under the age of 25 can’t afford period care products.* | Capital Daily  This newspaper article misrepresents finances as a barrier to the purchase of menstrual products as impacting 1 in 3 *Canadians* under 25 when it is 1 in 3 *young women* who are so affected since males do not menstruate.  <https://www.capitaldaily.ca/news/joni-menstrual-products> |
| **Dehumanising language and comparisons to men** | |
| Alexandria Ocasio-Cortez  *These anti choice bills… speaks from such a place of deep ignorance...it is ignorance that is hurting* ***people*** *across this country…When we talk about the law that was passed in Texas, we know that anti-choice bills are not about being pro-life…What this is about is controlling women’s bodies, and controlling people who are* ***not cisgender men.*** | CNN News  Alexandria Ocasio-Cortez from the Senate of the USA, in discussing legislation restricting women’s access to abortion, appears to refer to women as *not cisgender men*. An alternative explanation might be that by *not cisgender men* she means trans men but then *people who are* *not cisgender men* could also be reasonably be understood to include trans women and male non-binary people, making this an example of inappropriate inclusion as abortion is not something males might need.  <https://www.youtube.com/watch?v=GKBHcAhywOY> |
| Shimabukuro, T. T., Kim, S. Y., Myers, T. R., et al. (2021). Preliminary Findings of mRNA Covid-19 Vaccine Safety in Pregnant Persons. New England Journal of Medicine, *384*(24), 2273-2282.  *To identify* ***persons*** *who received one or both Covid-19 vaccine doses while pregnant or who became pregnant after Covid-19 vaccination, v-safe surveys include pregnancy questions for* ***persons who do not report their sex as male.*** | In this research on COVID-19 vaccination in pregnant women published in the New England Journal of Medicine, individuals to whom the questions on pregnancy were directed are described in the methods as being identified on the basis that they had indicated that they did not state their *sex as male* so presenting the male as the default human.  *https://www.nejm.org/doi/full/10.1056/nejmoa2104983* |
| *A Guide to Anal Sex*  *The appeal of anal sex when you* ***don’t have a prostate***  *Just because you* ***have a vagina*** *does not mean anal is off-limits. Many* ***vagina owners*** *love anal play…Anatomy of a* ***non-prostate owner*** | Teen Vogue  This article on anal sex for teenagers refers to young women as *vagina owners* and as *non-prostate owners*. In the latter instance, this usage accompanies a diagram of female anatomy including the vagina, uterus, cervix, urethra, and anus but omitting the illustration of, or naming of, the clitoris.  <https://teenvogue.com/story/anal-sex-what-you-need-to-know> |
| *American Cancer Society now recommends cervical cancer screening start at 25, not 21* ***Individuals with a cervix*** *are now recommended to start cervical cancers screening at 25 and continue through age 65, with the primary human papillomavirus (HPV) testing every five years as the preferred method of testing, according to a new guideline released Thursday by the American Cancer Society.* | CNN This article on cervical cancer screening describes women by referring to the presence or absence of their reproductive organs, as *individuals with a cervix* and *those who have had a hysterectomy*.  <https://edition.cnn.com/2020/07/30/health/new-cervical-cancer-screening-recommendations-wellness/index.html> |
| *Current Students of Strathcona Midwifery Collective*  *The midwifery model of care respects that each* ***birther*** *is a stakeholder in decision-making, and an expert on their own unique needs. I love that midwifery care holds space for traditional, cultural practices and evidence-based care. I view empowered* ***birthers*** *as the building blocks of healthy communities supported by compassionate, inclusive care providers.* | Strathcona Midwifery Collective  A midwifery student who is a part of this collective refers to pregnant and birthing women as *birthers* so reducing women to the process of giving birth.  <http://www.strathconamidwiferycollective.com/students> |
| *Dignified Menstruation: The* ***Dignity of Menstruators*** *throughout their Life*  *Dignified Menstruation examines the experiences of both* ***menstruators*** *and* ***non-menstruators*** *with respect to the dignity of menstruation in various circumstances. This book marks a milestone in changing the narrative of menstruation at the individual, family, society and global levels and thereby in achieving the goal of an equal society in which* ***menstruators*** *do not have to feel shamed by this most basic function of life.* | Amazon  This book and accompanying description, refers to *menstruators* and *non-menstruators*. It is unclear if *non-menstruators* means males or a mixed-sex group including girls who have not yet menstruated and postmenopausal women who no longer menstruate. Referring to women by a physiological process is considered demeaning.  <https://www.amazon.com.au/Dignified-Menstruation-Dignity-Menstruators-throughout/dp/9937672589> |
| Wilbur, J., Morrison, C., Iakavai, J. et al. (2021) “The weather is not good”: Exploring the menstrual health experiences of menstruators with and without disabilities in Vanuatu. *The Lancet Regional Health – Western Pacific* (2021). doi: 10.1016/j.lanwpc.2021.100325  ***Menstrual stigma*** *and inadequate water and private bathing facilities at home impacted all* ***menstruators*** *in the sample…This research provides further evidence about how disability and* ***menstruation stigma*** *and discrimination intersect to deepen inequalities*. | This research on disability and menstruation in remote Vanuatu uses the term *menstruators* to denote women and girls as a group (including those who have never menstruated or who have ceased menstruation). Not only does this term dehumanise by naming women and girls using a physiological process, but also by using a process that is stigmatised in their culture. The stigmatisation of menstruation is discussed at some length in the paper. There is a statement in the paper that the term *menstruator* is used to be inclusive, however the question must be asked inclusive to whom, the readers of the paper or the women themselves? The women quoted in the study refer to themselves as women and there is no indication that the term menstruator is in use in Vanuatu. Were the women in this study asked if they would like to be called *menstruators* or *women* or was it decided by the authors?  <https://www.thelancet.com/journals/lanwpc/article/PIIS2666-6065(21)00234-0/fulltext> |
| *Symptoms of breast pain in menopause*  *A* ***person with a uterus*** *reaches menopause after 12 months without a menstrual period. This stage follows a transitional period called perimenopause, where estrogen and progesterone levels in the body fluctuate unpredictably.* | Very Well Health  In this article on breast pain, women are referred to by reference to a reproductive organ or a reproductive stage as a *person with a uterus*, *perimenopausal people*, and *postmenopausal people*.  <https://www.verywellhealth.com/breast-pain-in-menopause-symptoms-5180788> |
| *Twitter@PPFA*  *"Sexually speaking,* ***folks with a vulva*** *are far more than reproductive machines."* | Planned Parenthood  In this tweet, Planned Parenthood unironically provide a quotation that states that women are more than *reproductive machines* but designate whom they are speaking of by referring to female genitalia using the phrase *folks with a vulva.*  https://twitter.com/PPFA/status/1476719759181983756 |
| Twitter@SandsUK  *Often the focus of support and comfort is on the* ***birthing parent****, which can leave partners or* ***non-birthing parents*** *feeling isolated and alone. Sands is here for you* | SANDS UK  The UK stillbirth and infant death charity SANDS, tweeted in Baby Loss Awareness Week using the terms *birthing parent* and *non-birthing parents.* This caused an uproar with many women distressed that *mother* was missing from the tweet. SANDS apologised stating that ‘bereaved mothers are mothers even if they don’t have a baby.’  <https://twitter.com/SandsUK/status/1314855702343680002> |
| *Highlights of prescribing information*  *DESCOVY is indicated in at-risk adults and adolescents weighing at least 35 kg for pre-exposure prophylaxis (PrEP) to reduce the risk of HIV-1 infection from sexual acquisition, excluding* ***individuals at risk from receptive vaginal sex.*** *Individuals must have a negative HIV-1 test immediately prior to initiating DESCOVY for HIV-1 PrEP. Limitations of Use (1.2): The indication does not include use of DESCOVY* ***in individuals at risk of HIV-1 from receptive vaginal sex*** *because effectiveness in this population has not been evaluated.* | USA Food and Drug Administration (USFDA)  The drug approval information from the USFDA states that this medication was approved for adults and adolescents but excluding individuals who have *receptive vaginal sex,* that is female people. So not only was drug approval limited to males, but women were described in reference to a sex act.  <https://www.accessdata.fda.gov/drugsatfda_docs/label/2019/208215s012lbl.pdf> |
| **Skewed emphasis** |  |
| ***GLANCE***  *“Since the global spread of the Coronavirus, and the introduction of measures against COVID-19, we saw many* ***parents*** *struggling with the very restricted or even prohibited access to the neonatal intensive care units due to the pandemic. They reached out to us, not only asking for help, but also for information if it was really necessary to keep* ***parents*** *apart from their newborns.”… To inform about consequences of* ***parent*** *and child separation, the campaign will shed light on the patients’ and the parents’ view by sharing experience reports from former patients, parents and other relatives.*  *Zero separation. Together for better care! Keep preterm and sick babies close to their* ***parents.*** | Global Alliance for Newborn Care  This webpage describes a campaign promoting the wellbeing of premature infants. It makes reference to the COVID-19 pandemic which saw premature infants separated from their mothers in unprecedented numbers as Neonatal Intensive Care Unit (NICU) visiting policies greatly restricted access to the Units. During the pandemic, visiting policies allowing only a single visitor per day that referred to *parents*, and did not differentiate between fathers and mothers or prioritise mothers, resulted in increased mother-infant separation. Such policies did not reflect that the consequences of mother-infant and father-infant separation are vastly different and that both parents are not equally important to newborns. They did not recognise that if parental access to infants is restricted, the best interests of the child require that maternal access be prioritised. In the context of scarcity of access to NICUs, advocacy should focus on the importance of *mothers* to infants. However, this campaign focusses on keeping *parents* *and infants* together in a shift that has also been reflected in the 2021 theme for World Prematurity Day which is ‘Zero Separation Act now! Keep *parents and babies* born too soon together.’  <https://www.glance-network.org/news/details/zero-separation-global-campaign> |
| **Inappropriate exclusion** |  |
| *Barring* ***cisgender women*** *from the Descovy trials was a bad call*  *Of the estimated 38 million people living with HIV around the world, more than half are women. Women, especially young women, are at greater risk for HIV infection than men. So why would a company omit* ***cisgender women*** *from its clinical trials testing a pre-exposure prophylaxis (PrEP) regimen?* | Stat News  This online news website describes the omission of women from clinical trials of an HIV prevention drug. However, rather than using a sexed definition of the word *women*, it applies a gendered definition and incorrectly confines the group who were omitted from the research to women in a gendered sense by using the phrase *cisgender women*. *Cisgender women* means females with the gender identity of *woman*. By implication, this terminology inappropriately suggests that women who do not identify as *cisgender women* (such as transgender men or female nonbinary people, as well as females who do not have a gender identity) may have been included in these trials when in fact all females, regardless of their gender identity, were excluded.  https://www.statnews.com/2019/11/25/descovy-trials-excluded-cisgender-women-bad-call/#:~:text=Of%20the%20estimated%2038,for%20HIV%20infection%20than%20men. |
| **Asynchrony between how women and men are referred to** |  |
| *Everything you need to know about HPV in* ***vulva owners*** (15/3/21)  Does it only affect **individuals who have a vagina**? *Human Papillomavirus (HPV) in Men* (15/3/21) *Most* ***men*** *with HPV never experience symptoms or realize that they have the infection.* | Healthline  These two articles on Human Papilloma Virus (HPV) published on the Healthline website were reviewed on the same date. The article on HPV in women is entirely desexed with women referred to in relation to body parts as *vulva owners* and *individuals with a vagina* and with the word *women* absent. In contrast, the article on HPV in men uses sexed terms, including *men* and *women* throughout.  <https://www.healthline.com/health/healthy-sex/hpv-in-women> **[https://www.healthline.com/health/sexually-transmitted-diseases/hpv-in-men#symptoms](https://www.healthline.com/health/sexually-transmitted-diseases/hpv-in-men#symptoms)** |
| *Mission and Vision*  *Uninhibited has de-stigmatised menstrual, sexual and reproductive health for 200,000+* ***marginalised menstruators*** *in 10 states across India. We’ve seen* ***menstruators*** *seek healthcare and support, reclaim spaces at school, workplaces and communities, eventually leading to reduced gender equity and better health and support. Support our journey to reach 2.5 million* ***menstruators, men and boys*** *by 2025.* | Uninhibited  The mission and vision of Uninhibited describes women and girls using the dehumanising term *menstruators* yet does not apply dehumanising terminology to males who are referred to as *men* and *boys.*  <https://uninhibited.org.in/our-journey/> |
| *Medical Definition of male (29/3/21)* *The sex that produces spermatozoa.* *Medical Definition of Female (29/3/21) The traditional definition of female was "an individual of the sex that bears young" or "that produces ova or eggs". However, things are not so simple today. Female can be defined by physical appearance, by chromosome constitution (see Female chromosome complement), or by gender identification. Female chromosome complement: The large majority of females have a 46, XX chromosome complement (46 chromosomes including two X chromosomes). A minority of females have other chromosome constitutions such as 45,X (45 chromosomes including only one X chromosome) and 47,XXX (47 chromosomes including three X chromosomes).* | RxList  This online medical encyclopedia for pharmacists provides definitions of *male* and *female* that were updated on the same date by the same editor. The definition of *male* is straightforward and scientifically conventional whereas the definition of *female* has seemingly taken on Queer Theory ideas regarding sex as a spectrum and is complex and unclear.  <https://www.rxlist.com/male/definition.htm>  <https://www.rxlist.com/female/definition.htm> |
| **Making women invisible where femaleness is central** | |
| *Clinical Competencies for the Practice of International Board Certified Lactation Consultants*  *The IBCLC has the duty to act with reasonable diligence and will: 1. Assist* ***clients*** ***and families*** *with decisions regarding feeding their child(ren) by providing evidence-based information that is free of any conflicts of interest.* | International Board of Lactation Consultant Examiners  These clinical competencies for the international qualification of International Board Certified Lactation Consultant, a role centred on supporting mothers to breastfeed their babies is entirely missing the words *women* and *mothers*.  <https://iblce.org/wp-content/uploads/2018/12/clinical-competencies-2018.pdf> |
| Twitter@USbreastfeeding  *Pregnant and breastfeeding* ***families****, health care providers, and all stakeholders are urged to share how the pandemic is impacting* ***families****. Your insight will guide our work to create a landscape of breastfeeding support, during this pandemic and beyond.* | United States Breastfeeding Committee (USBC)  This tweet from USBC is regarding how breastfeeding was adversely impacted by the COVID-19 pandemic. In the US, the major health organisations initially recommended that mothers and infants be separated if mothers had COVID-19. This had a devastating impact on maternal mental health and the ability of women to breastfeed. Nonetheless, this tweet refers only to the pandemic impacting *families.*  <https://twitter.com/USbreastfeeding/status/1324846536820465666> |
| Williams, C. R., Huff, A., & Meier, B. M. (2021). Dissident blood: Using critical feminist study to advance the health and human rights of menstruators. *Health and Human Rights, 23*(1), 293-296.  ***Half of humanity has personal experience with menstruation****, spending approximately half of their lives managing their menstruation, yet it is only recently that “menstrual health” has received more than passing attention in health policy...The Palgrave Handbook of Critical Menstruation Studies seeks to codify the field, exploring the meaning of menstruation to* ***menstruators*** *and examining menstrual health within diverse social and cultural contexts…Menstruation impacts a wide range of human rights, from the right to education to the right to participation in cultural life, and promoting the individual* ***dignity of menstruators*** *ensures that their right to exercise meaningful agency is protected under international law*. | In this paper, note is made that menstruation is something that half the population experiences and that it is largely absent from policy. Which half of the population experiences menstruation is not mentioned. In addition, it is not identified that menstruation is not appropriately considered in policy because it is a condition impacting women and their situation and needs are underrepresented in policy generally. That is, this absence is a reflection of sexism. While using dehumanising language, the right to dignity of girls and women who menstruate is stated apparently without irony.  <https://www.ncbi.nlm.nih.gov/pmc/articles/PMC8233013/> |
| *What We Fear in the Wake of the Texas Abortion Law*  *Whether this barrier to health care is temporary or permanent, one thing we know for certain is that* ***people who decide to end their pregnancies*** *will now be doing so later in gestation—either because of waiting for legal abortion to return in Texas or needing to book out-of-state visits weeks in advance. These delays will increase health risks as well as financial burdens and lead more* ***people*** *to take legal risks to terminate. What they will not do, for the large part, is make* ***Texans*** *and others affected in the region decide they should just give birth.* | Time Magazine  In this article about abortion law changes in Texas, USA, women are described as *patients*, *people* and *Texans*. There is no indication that these legislative changes impact only women so marginalising them.  <https://time.com/6101739/abortion-access-alabama/> |
| Timeline to eliminate cervical cancer varies widely based on wealth More than 90 percent of cervical cancers can be traced to HPV infection, and approximately **14,000 people** are diagnosed with cervical cancer per year in the United States alone — which leads to roughly 4,000 deaths annually. | Gillings School News  In this article about cervical cancer, readers are informed that 14 000 *people* are diagnosed with cervical cancer each year of which 4000 die and it is not identified that in all of these cases, those with cancer are female.  <https://sph.unc.edu/sph-news/timeline-to-eliminate-cervical-cancer-varies-widely-based-on-wealth/> |
| *Menopause at work*  *The menopause is a natural stage of life which* ***affects around half of the population****. The menopause usually happens between 45 and 55 years of age but it can also happen earlier or later in* ***someone's life****. For many* ***people*** *symptoms last about 4 years, but in some cases symptoms can last a lot longer…Some* ***people*** *might also experience early menopause or go through surgical menopause earlier in their lives. These types of menopause can be medically complicated, so employers should consider this when supporting their* ***staff…*** *It's important for employers to be aware that the* ***menopause and its symptoms can affect any of their staff at any time****, including:*   - *those going through the menopause* - *relatives, colleagues and carers who are supporting someone going through it* - *trans people – 'trans' is an umbrella term used to describe people whose gender is not the same as the sex they were assigned at birth* - *intersex people*   *If an employee or worker is put at a disadvantage or treated less favourably because of their menopause symptoms, this* ***could be discriminatory if connected to a protected characteristic.*** | Advisory, Conciliation and Arbitration Service.  This information on menopause for employers neglects to state that it is women who experience this physiological state. It is noted that this process impacts half the population but there is no information on what half of the population is affected. Further confusion is added by a statement that menopause can impact any staff member. Reference is made to the fact that treating someone less favourably because of menopause symptoms could be discriminatory if it is connected to a protected characteristic without noting that the protected characteristic impacting those experiencing menopause is *sex* and specifically that all those who experience menopause are female.  <https://www.acas.org.uk/menopause-at-work> |
| *Care for Breastfeeding Women (5/5/20)* *Whether and how to start or continue breastfeeding should be determined by the* ***mother,*** *in coordination with her family and healthcare providers. A* ***mother*** *with suspected or confirmed COVID-19 should be counseled to take all possible precautions to avoid spreading the virus to her infant.* *Care for Breastfeeding Women (16/11/20)****Breastfeeding person*** *has suspected or confirmed COVID-19, but breastfed child does not have COVID-19* ***Isolation and quarantine***   - *The* ***breastfeeding person*** *with suspected or confirmed COVID-19 should follow information on home isolation.* - *A child being breastfed by* ***someone*** *with suspected or confirmed COVID-19 should be considered as a close contact of a person with COVID-19, and should be quarantined for the duration of the* ***lactating parent’s*** *recommended period of home isolation and 14 days thereafter.*  *Care for Breastfeeding People (29/1/21)****Breastfeeding person*** *has suspected or confirmed COVID-19, but breastfed child does not have COVID-19* ***Isolation and quarantine***   - *The* ***breastfeeding person*** *with suspected or confirmed COVID-19 should follow information on home isolation.*  *A child being breastfed by someone with suspected or confirmed COVID-19 should be considered as a close contact of a person with COVID-19, and should be quarantined for the duration of the lactating parent’s recommended period of home isolation and 14 days thereafter.* | United States Centers for Disease Control COVID-19  From November 2020 to January 2021 the United States Centers for Disease Control published three versions of their Guidance on Breastfeeding during the COVID-19, progressively desexing the text with the terms *woman*, *women*, mother, and *mothers* replaced with the terms *people*, *person*, *lactating person*, *lactating parent*, and *someone*. This obscured that this guidance impacted upon women and the unique relationship between the mother and her infant. Such usage risks jeopardising successful communication with lower literacy groups and US residents for whom English is not their first language. [**https://www.cdc.gov/coronavirus/2019-ncov/hcp/care-for-breastfeeding-women.html**](https://www.cdc.gov/coronavirus/2019-ncov/hcp/care-for-breastfeeding-women.html) |
| ACC invest $44.9 million in sexual violence primary prevention, tender open, report on cost of sexual violence  *Minister for ACC Carmel Sepuloni announced the funding investment noting that the sexual violence primary prevention system would focus on more than just violence, including the whole community and addressing the social drivers that allow sexual violence to occur.* | New Zealand Family Violence Clearing House  This advertisement of tender and the associated tender proposal request on prevention of sexual violence in Aotearoa New Zealand do not contain the words *women* or *men.* This is despite the fact that the biggest risk factor for being a victim of sexual violence is being female and that perpetrators of sexual violence are overwhelmingly male. Sexual violence appears almost as a disembodied phenomenon that is independent of human beings.  <https://nzfvc.org.nz/node/4381> |
| *Scottish Government campaign in push for people to attend smear tests* *A NEW campaign has been launched urging* ***people who are eligible for a smear test*** *to attend when invited.*  *The Scottish Government campaign encourages* ***people*** *not to ignore their invite – even if they think everything is fine – and highlights that* ***two people*** *die from cervical cancer in the UK every day.* | The National  This newspaper article reports on a campaign by the Scottish Government to encourage cervical cancer screening that notes that two *people* a day die from cervical cancer in the UK. It does not say that it is *women* who are at risk for cervical cancer and it is very unclear who the *people* are who need a smear test.  <https://www.thenational.scot/news/19637980.scottish-government-campaign-push-people-attend-smear-tests/> |
| *Sex of Fetus Affects Immune Response to COVID-19 During Pregnancy* ***Male placentas*** *produce more proinflammatory molecules than* ***female placentas****, while* ***people*** *carrying* ***male fetuses*** *produce fewer antibodies in response to infection, a study finds.* ***Pregnant people*** *respond to COVID-19 differently depending on the sex of their unborn child, according to a study released yesterday (October 19) in* [Science Translational Medicine](https://www.science.org/doi/10.1126/scitranslmed.abi7428)*.* ***Male placentas*** *produced more proinflammatory genes and proteins than* ***female placentas*** *after the* ***parent*** *contracted COVID-19, and* ***people gestating sons*** *produced fewer antibodies following infection. They also passed fewer protective antibodies on to the fetus.* | The Scientist  This magazine article reports on a study on COVID-19 during pregnancy which found that the sex of the fetus impacted maternal response to infection. The text is desexed in relation to the pregnant women but not in relation to the fetuses that women are pregnant with. Thus, fetuses and placentas are *male* and *female* but those who are pregnant are *persons* or *people*. This mix of sexed and desexed language is counter intuitive. Why is referring to the sex of fetuses acceptable but referring to the sex of the pregnant woman unacceptable? The language regarding pregnant women may have been desexed in order to account for those who have a gender identity meaning that they do not identify as women, but when the article is discussing the impact of sex, and the uniquely female state of pregnancy, avoidance of sexed language reduces clarity in what is an already complex story. For example, when *male placenta* is said in the same sentence as *pregnant people*, the possibility is raised that a gendered understanding of words is being prioritised throughout the article and that *male placentas* might mean placentas of pregnant trans men when this is not the case at all. [**https://www.the-scientist.com/news-opinion/sex-of-fetus-affects-immune-response-to-covid-19-during-pregnancy-69329**](https://www.the-scientist.com/news-opinion/sex-of-fetus-affects-immune-response-to-covid-19-during-pregnancy-69329) |
| Twitter@JoeBiden  *Texas law SB8 will significantly impair* ***people’s*** *access to the health care they need—particularly for communities of color and* ***individuals*** *with low incomes. We are deeply committed to the constitutional right established in Roe v. Wade and will protect and defend that right.* | President of the USA, Joe Biden  This tweet from the President of the USA on legislation restricting women’s access to abortion refers only to this impacting on *people* and makes no reference to this being something that is impacting on the constitutional rights of *women*.  <https://twitter.com/joebiden/status/1433183957424689157> |
| **Confusing terminology** | |
| Brandt, J. S., Patel, A. J., Marshall, I., & Bachmann, G. A. (2019). Transgender men, pregnancy, and the ‘new’ advanced paternal age: A review of the literature. *Maturitas, 128*, 17-21.  *In this review, we discuss the obstetrical care of transgender men with* ***advanced paternal age****… we were forced to extrapolate data from studies about cisgender women with advanced maternal age as there was limited data on the pregnant transgender man with* ***advanced paternal age****.* | This paper suggests creation of a new term for the situation of pregnant transgender men over the age of 35 years being the new *advanced paternal age*. There is no appreciation of the confusion that could be caused by the use of *paternal* in the clinical or research setting to apply to females, nor that there are specific, different, risks with advanced male age.  *https://pubmed.ncbi.nlm.nih.gov/31561817/* |
| *Gender Inclusive Language in Perinatal Services: Mission Statement and Rationale*  *Previous example: “Maternity care should be available to all”*  *New example: “Perinatal care should be available to all”* | Brighton and Sussex University Hospitals National Health Service (NHS) Trust  This guide for a UK health service suggests that *maternity care* can be replaced with *perinatal care* but these terms have different meanings. *Maternity care* focuses on the woman and starts from the booking into the maternity system during first trimester and continues even where there is fetal or infant demise. *Perinatal care* is focussed on the fetus/infant and starts at fetal viability at about 24 weeks gestation and until 7 or 28 days after birth. The guide from Brighton and Sussex NHS Trust has some text suggesting some understanding that maternity and perinatal mean different things. However, the example supplied does not reflect this and there is no acknowledgement of the potential impact of shifting from focusing on women to fetuses/infants by changing terms.  <https://www.bsuh.nhs.uk/maternity/wp-content/uploads/sites/7/2021/01/Gender-inclusive-language-in-perinatal-services.pdf> |
| **Unclear text descriptions for the visually impaired** |  |
| Facebook: @LaLecheLeagueUSA  *[Image: Photo of crying baby being held by one person while crying while another person tries to comfort them. Text: I'm not ready to leave my baby with relatives. Is that normal?]* | La Leche League USA  This Facebook post includes an image description for the visually impaired. Image descriptions aim to provide the same or equivalent information that a sighted person would get when they look at the picture. This picture shows a woman holding a baby who is being comforted by a man however, a visually impaired person is not provided with this information because the text refers only to a *person* holding the baby and a *person* comforting them.  <https://www.facebook.com/LaLecheLeagueUSA/posts/2999865906708602> |
| **Studies where sex is not considered but should have been** | |
| Veale, J., Watson, R. J., Adjei, J., & Saewyc, E. (2016). Prevalence of pregnancy involvement among Canadian transgender youth and its relation to mental health, sexual health, and gender identity. *International Journal of Transgenderism, 17*(3-4), 107-113.  *To assess pregnancy involvement, an item asked youth, “****How many times have you been pregnant or gotten someone pregnant****?”…Five hundred-forty trans youth responded to a question about pregnancy involvement, and 111 skipped this question because they reported they had never had sex. Of these****, 26 reported pregnancy involvement*** *at least once: 22* ***youth*** *(3%) reported involvement one time, and 4* ***youth*** *(1%) reported two or more times. Five* ***youth*** *(1%) answered that they were not sure.* | This study on *pregnancy involvement* (defined as getting pregnant or getting someone pregnant) on the mental and sexual health of transgender young people had a mixed sex group of respondents but did not report data disaggregated by sex even though *pregnancy involvement* is very different for males in comparison to females and that this would likely impact the mental and sexual health of individuals. |
| Scala, M., Marchman, V. A., Brignoni-Pérez, E., Morales, M. C., Dubner, S. E., & Travis, K. E. (2021). Impact of the COVID-19 pandemic on developmental care practices for infants born preterm. *Early Human Development, 163*, 105483  *During that period, visitation was limited to* ***parents*** *only. On March 30, 2020,* ***only one parent*** *was allowed to visit per infant for the entire hospital stay. By May 7, 2020,* ***parents*** *could alternate days visiting but could not come together…* ***Parents*** *in the COVID-19 period visited their infants roughly half as often as in the preceding year… Although reduction in* ***family visitation*** *is not an unexpected finding considering the changes to hospital policies, it represents a significant threat to* ***parent-infant bonding****, the delivery of important* ***parent-delivered*** *care activities, and positive health outcomes for* ***parents*** *and infants* | This paper did not differentiate between the access of infants in the NICU to their mother versus their father and other family members. While maternal-infant separation is known to have an adverse impact on maternal caregiving capacity, mental health and breastfeeding, the impact of separation from mothers separate from fathers and other family members was not considered.  *https://pubmed.ncbi.nlm.nih.gov/29321720/* |
| *Consultation on Coronial Investigation of Stillbirths: Consultation Document*  *The stillbirth of a baby is a distressing experience for* ***parents and families.*** *Bereaved* ***parents*** *will want to know why their baby was not born alive.* | Government of the United Kingdom  This consultation on coronial investigation of stillbirths in the UK did not differentiate between the experiences of mothers and fathers despite the physical experience being very different and research indicating differences in the emotional and psychological response to stillbirth in men and women.  <https://consult.justice.gov.uk/digital-communications/coronial-investigations-of-stillbirths/> |
| **Research where a mix of sex and gendered understanding of words prevents assessing findings by sex** | |
| Messick, C. A. (2020). Treatment efficacy for human papillomavirus-related anal squamous cell dysplasia in an under-represented population: human immunodeficiency-negative, **non-men** having sex with **men**, and non-transplant population. *Colorectal Disease, 22*(1), 29-35.  There were 41 patients identified as meeting the inclusion criteria, 34 (83%) of whom were **women**… This study reports treatment outcomes for HPV-related anal dysplasia in an HIV-negative, nonimmune-suppressed, **non-MSM [non-men having sex with men]** population. | In this paper, *non-men* appears to describe a mixed sex group and while the demographic information provided states the number of women in the study, they do not make clear whether a sexed or gendered meaning is being applied nor provide any information on the number of men in the study. This lack of clear disaggregation makes it impossible to determine the numbers of males and females in the study and to understand the impact of sex on outcomes.  https://pubmed.ncbi.nlm.nih.gov/31344295/ |
| McMillan, T. M., Aslam, H., Crowe, E., et al. (2021). Associations between significant head injury and persisting disability and violent crime in women in prison in Scotland, UK: a cross-sectional study. *The Lancet Psychiatry, 8*(6), 512-520.  <https://doi.org/10.1016/S2215-0366(21)00082-1>  *In this cross-sectional study,* ***female offenders*** *were recruited between Feb 2, 2018, and Sept 30, 2019, from four prisons across Scotland, UK. Of the* ***355 women*** *incarcerated in the four prisons we recruited from, 109 (31%) expressed an interest in being part of the study and were seen by researchers, and all were deemed eligible to take part. Five of these individuals identified as* ***transgender women****.* | This paper describes research on head injury and violent offending in women in Scottish prisons. While the method specifies that *female* offenders were recruited, the results identifies that about 5% of the study sample were trans women (i.e. male). Results in this study are not disaggregated by sex despite male sex being the strongest predictor of violent crime and it being unknown how gender identity impacts criminal activity. A letter to the editor noting this and suggesting that results could be skewed by a mixed sex sample was initially accepted for publication by the journal but was then rejected.  <https://twitter.com/JoPhoenix1/status/1430843098427428866?s=20> |
| **Anatomical inaccuracy** | |
| ***Breast/Chest*** *Cancer Screening for* ***Breast Owners***  *It is important to be aware of how your* ***breast/chest*** *looks and to tell your health care provider if you notice any changes…At age 25, you should learn* ***breast/chest*** *self-awareness and how to do a monthly* ***breast/chest*** *self-exam.* | Ohio State University Wexner Medical Center  This factsheet from a medical service refers to breast/chest cancer screening seemingly erroneously suggesting that breast cancer and chest cancer are the same thing.  <https://healthsystem.osumc.edu/pteduc/docs/BreastChestCanScreenBreastOwners.pdf> |
| *As a trans woman, do I need to get screened for cervical cancer?*  *If you’re a trans woman, you may not have given much thought to Pap tests and cervical cancer. And if you haven’t, that makes a fair amount of sense. After all,* ***in order to get cervical cancer, you need to have a cervix*** *— that is, the organ that connects the vagina to the uterus….If you’re a trans woman and have not had bottom surgery, you aren’t at risk for cervical cancer. If, however, you’re a trans woman who has had bottom surgery to create a vagina (vaginoplasty) and possibly a cervix, there’s a very small risk that you can develop cancer in the tissues of your neo-vagina or* ***neo-cervix****…It can be difficult to make cancer screening a priority, especially when there’s not a lot of information out there about cervical cancer risks for trans women. You may also be concerned about things like experiencing transphobia during the screening process. Maybe you feel you have more pressing health concerns. Or maybe you just don’t want to think about cancer screening. Still, it’s important to take care of your health by* ***getting the cancer screening you need.*** *Screening means checking for cancer before there are any symptoms. Here’s the bottom line: if you’re a trans woman who’s had bottom surgery, discuss your personal risk for cancer in your neo-vagina or* ***neo-cervix*** *with your healthcare provider, and come up with a plan for cancer screening that works for you.* | Canadian Cancer Society  This webpage from a cancer advocacy organisation provides information for trans women on cervical cancer screening. It erroneously implies that surgery can create a cervix or cervix-analogous organ and that cervical cancer screening is a procedure that trans women might have a reason to undergo. This suggestion might place health professionals approached by trans women at risk of accusations of bigotry for not being willing to provide an unnecessary or impossible procedure. It could also increase confusion around what a cervix is, particularly for women who need cervical cancer screening and who may not know what the cervix is and be unaware that the cervix is a female reproductive organ. In contrast, the UK Cancer advocacy organisation provides clear and accurate information for trans women on their anatomy and lack of need for cervical cancer screening.  <https://cancer.ca/en/cancer-information/find-cancer-early/screening-in-lgbtq-communities/as-a-trans-woman-do-i-need-to-get-screened-for-cervical-cancer>  https://www.cancerresearchuk.org/about-cancer/cancer-symptoms/spot-cancer-early/screening/trans-and-non-binary-cancer-screening |
| **Confusion about who is included and who is not in a definition** | |
| World Health Organization, Human Reproduction Programme, UNAIDS, UNFPA, & UN Office of the Commissioner for Human Rights. (2017). *Consolidated Guideline on Sexual and Reproductive Health and Rights of Women Living with HIV*. Geneva: World Health Organization.  *This guideline is intended to address* ***women living with HIV in all their diversity****, including, but not limited to:* ***women who are*** *heterosexual, lesbian, bisexual,* ***transgender*** *or intersex; women who use or have used drugs; women who are or have been involved in sex work; women who are single, married or in stable relationships, separated, divorced or widowed; women who are and are not sexually active; women and girls who have undergone female genital mutilation (FGM); women who have tuberculosis (TB), malaria, hepatitis B or C and/ or other co-morbidities; women who are currently or have previously been incarcerated, detained or homeless; women who are economic or political migrants; women who are indigenous; women living with disabilities; as well as adolescent girls who have acquired HIV perinatally, in childhood or during adolescence.*  *Key considerations on cervical cancer for* ***transgender men*** *living with HIV  It is important to counsel* ***transgender women*** *who use oral contraceptive pills for feminization about the higher risk of thrombotic events with ethinyl estradiol than with 17-beta estradiol.* | This guidance from WHO on the sexual and reproductive health of *women with HIV* includes a definition of who is covered by the guidance. Unfortunately, the definition of *women living with HIV in all their diversity* is unclear as the word *women* appears to hold both sexed and gendered meanings. The application of a gendered meaning of women is made explicit in that *transgender women* are specifically listed as included in the guidance. One would think that this gendered meaning of *women* would preclude inclusion of transgender men, however text on page 78 of the document shows that transgender men are in fact included. Thus, the *women living with HIV in all their diversity* appears to be a female cohort regardless of gender identity with the addition of transgender women but this is not made explicit. |
| **Application of a gendered understanding of ‘male’ and ‘female’** |  |
| Margaria, A. (2020). Trans men giving birth and reflections on fatherhood: what to expect? *International Journal of Law, Policy and the Family, 34*(3), 225-246.  *In the context of trans procreative rights, trans masculine practices of pregnancy and birth have attracted wide media coverage and sparked ‘moral panic’. This is, inter alia, due to their visible departure from the conventional imaginary of reproduction that portrays* ***‘male’*** *and* ***‘female’*** *contributions as clearly defined and distinct from one another and, in particular, to the challenges they pose to gendered notions of pregnancy… Moreover, to claim that McConnell’s registration as* ***‘mother’*** *does not necessarily indicate that he is* ***female*** *– as English courts explicitly did – clashes with the social reality that almost everyone who McConnell shall meet will understand his status as* **‘mother’** *in traditionally* ***gendered terms****.* | This paper discusses controversy over trans men who have given birth to children wanting to be registered on their children’s birth certificate as fathers rather than mothers. It appears to apply a gendered understanding of the words *male* and *female,* seems to imply that the roles of *males* and *females* in human reproduction are not fixed and that in stating that it is only females who become pregnant, one is *gendering* pregnancy rather than recognising pregnancy as an inherently sexed process. The UK legal understanding of *mother* as a term that applies to the sexed role of *female parent* is criticised. While the paper argues for the *degendering* of parenthood the author is in fact arguing for a gendered understanding of motherhood and fatherhood to be prioritised over a sexed understanding.  <https://doi.org/10.1093/lawfam/ebaa007> |
| *Transgender man gives birth to non-binary partner’s baby with* ***female sperm donor***  *The 39-year-old transitioned to a man 12 years ago. But he still had maternal instincts and six years ago stopped taking testosterone in the hope of one day having a child. And that dream came true when he and partner Jay had a bouncing baby. Jay is non-binary- so does not identify as male or female. The sperm donor was a trans woman.* | The Mirror  This newspaper article, describes a trans woman who donated sperm to enable the conception of a baby as a *female sperm donor* so inappropriately using the term female to apply to gender identity.  <https://www.mirror.co.uk/news/uk-news/transgender-man-gives-birth-non-21177808> |
| ***Altering quotations*** |  |
| *Using Cannabis While Breastfeeding May Not Be as Harmful to Infants as Once Thought, According to New Study*  *"****[Pregnant people]*** *were getting mixed messages. Their OBs would approve marijuana for nausea, and we, as pediatricians, would make them stop. This study is encouraging to know that concert data will help guide recommendations but still cautious moving forward." The biggest concern we heard from medical doctors about breast milk health is not about THC exposure but essential nutrition and health of the birther or person chestfeeding."The number one thing I see in my practice is that* ***[birthers]*** *are not eating and not taking enough nutrition in for themselves," says Dr. Patel.* | Parents Magazine  In this magazine article, the words of a doctor who is quoted are altered to seemingly replace the phrase *pregnant women* with *pregnant people* and *birthers* as indicated by the use of square brackets. Such substitution is disrespectful of the person quoted who may object to the use of the terms substituted for their own.  [https://www.parents.com/news/cannabis-while-breastfeeding-study](https://www.parents.com/news/cannabis-while-breastfeeding-study/?utm_medium=browser&utm_source=parents.com&utm_content=20211116&utm_campaign=1604718&fbclid=IwAR0xEhwPa_uHBGeoA_Shbd3IpuYSrS31B-BlHcZVYdy-udzT0eqz0JO0wIo) |
| **Historical revisionism** | |
| 1993- Nomination of Ruth Bader Ginsburg, to be Associate Justice of the Supreme Court of the United States  *The decision whether or not to bear a child is central to a* ***woman’s*** *life, to* ***her*** *well-being and dignity. It is a decision she must make for* ***herself****. When Government controls that decision for* ***her****,* ***she*** *is being treated as less than a fully adult human responsible for* ***her*** *own choices.*  2021-Twitter@UCLA  *The decision whether or not to bear a child is central to a* ***[person’s]*** *life, to* ***[their]*** *well-being and dignity…When the government controls that decision for* ***[people]****,* ***[they are]*** *being treated as less than a fully adult human responsible for* ***[their]*** *own choices.* | United States Civil Liberties Union  In 1993 Ruth Bader Ginsberg, made a statement regarding the reproductive rights of women during her nomination hearing to be an Associate Justice of the Supreme Court of the United States. In 2021, on the first anniversary of her death, this statement was desexed by the American Civil Liberties Union with the words *woman*, *her*, and *she* being replaced with *people*, *they* and *their*.  <https://www.govinfo.gov/content/pkg/GPO-CHRG-GINSBURG/pdf/GPO-CHRG-GINSBURG.pdf>  <https://twitter.com/aclu/status/1439259891064004610> |
| Caughey, A. B., Krist, A. H., Wolff, T. A., et al. (2021). USPSTF Approach to addressing sex and gender when making recommendations for clinical preventive services. *JAMA*.  *To advance its methods, the USPSTF reviewed its past recommendations that included the use of sex and gender terms, reviewed the approaches of other guideline-making bodies, and pilot tested strategies to address sex and gender diversity…The current language in many USPSTF recommendations lacks clarity and uses sex and gender terms inconsistently and interchangeably. For example, the USPSTF recommendation statement for breast cancer screening states that it “applies to…****women*** *aged 40 years or older.” However, it is unclear how or whether it applies to transgender, gender nonbinary, or gender nonconforming persons assigned female sex at birth or intersex individuals with breasts…The USPSTF most often used* ***gender terms****, referring to* ***men*** *or* ***women****, instead of the sex terminology of* ***male*** *and* ***female****.* | This paper from the United States Preventative Service Task Force describes the findings of their review of the organisation’s past recommendations regarding sex and gender identity. They say that their previous recommendations have been unclear because they have used sexed and gendered terms interchangeably. The sexed terms they are referring to here are *male* and *female* and the gendered terms are *men* and *women*. However, past recommendations used the terms *women* and *men* in a wholly sexed sense and so there was no mixing of sexed and gendered terms, only a sexed usage of *women* and *men*. It is not recognised that it is only recently that new gendered conceptualisations of the words *women* and *men* have arisen and that this is what has caused confusion. This paper also reflects the erroneous Queer Theory understanding of individuals with intersex variations as being of a third sex.  <https://doi.org/10.1001/jama.2021.15731> |
| **Proposed or enacted legislation making sex invisible** |  |
| *Ministerial and Other* ***Maternity Allowances*** *Bill*  *Payment of* ***maternity allowance****: Ministerial office*  *(1) A* ***person*** *designated as a Minister on Leave under this section is to be paid an allowance in accordance with section 2.* *Ministerial and Other* ***Maternity Allowances*** *Act**Payment of* ***maternity allowance****: Ministerial office**1)* ***A mother or expectant mother*** *designated as a Minister on Leave under this section is to be paid an allowance in accordance with section 2.* | Parliament of the United Kingdom  The Ministerial and Other Maternity Allowances Bill was to allow government ministers in the British Parliament to take maternity leave when they gave birth. As originally introduced, this Bill was desexed apart from reference to *maternity* in the name. During the discussion of the Bill, the issue of it being desexed was raised, extensive debate and discussion occurred in the House of Lords on this issue, and amendments were made so that the final Act refers to *mothers* and *expectant mothers* instead of *people*.  <https://researchbriefings.files.parliament.uk/documents/LLN-2021-0004/LLN-2021-0004.pdf>  <https://www.legislation.gov.uk/ukpga/2021/5/section/1/enacted> |
| *NSW Abortion Law Reform Act 2019*  *7.Requirement for information about counselling*  *(1) Before* ***performing a termination on a person*** *under section 5, a medical practitioner must—*  *(a) assess whether or not it would be beneficial to discuss with the* ***person*** *accessing counselling about the proposed termination, and* ***(****b) if, in the medical practitioner’s assessment, it would be beneficial and the person is interested in accessing counselling, provide all necessary information to the person about access to counselling, including publicly-funded counselling.* | Parliament of New South Wales, AustraliaThe NSW Abortion Law Reform Act 2019 legalised abortion in NSW, Australia. However, it was enacted in a desexed form in which the word woman has been replaced with *person*. This obscures the inherently sexed state of pregnancy and decisions concerning pregnancy termination. <https://legislation.nsw.gov.au/view/html/inforce/current/act-2019-011> |
| **Gendered interpretation of words undermining the purpose of policies** | |
| *Donation if you are transgender*  *NHS Blood and Transplant considers* ***all donors to be the sex and/or gender that they identify*** *as, including nonbinary, genderfluid and agender donors…As with all donors, a careful and sympathetic consideration of sexual risk factors is undertaken. The* ***deferral for men who have sex with men applies to men only, regardless of whether you are cis or trans. It would not apply to you if you are a transgender woman*** *or if you are a person of another gender or no gender.* | NHS Blood and Transplant  The NHS blood donation service has a risk management policy excluding *men* who have had sex with other *men* from donating blood within three months of this sexual activity. However, for at least a year the NHS website was clear that they were applying a gendered understanding rather than a sexed understanding of *men* in this policy. That meant that donation deferral did not apply to males with a gender identity of woman (i.e. transgender women). In addition, females with a gender identity of man who had sex with males or other females with the gender identity of man (i.e. transgender men) were captured by this policy and ineligible to donate blood within the deferral period as they were considered to be *men who have sex with men*. This gendered interpretation of the word *men* undermined a risk management policy that was based on an issue related to the sexed body of males rather than their gender identity. It worked against the intent of the policy which was to facilitate safe blood donation. The text of the webpage has since been changed. It no longer refers to men who have sex with me but simply states that ‘As with all donors, a careful and sympathetic consideration of sexual risk factors is undertaken.’  <https://web.archive.org/web/20210415145201/https://www.nhsbt.nhs.uk/who-we-are/our-staff/lgbtplus-network/donation-if-you-are-lgbtplus/> |
| *Male abuser denied help because lover is non-gender*  ***A man convicted of attacking his non-binary partner*** *has been prevented from enrolling in a domestic abuse rehabilitation scheme because it is for heterosexual couples…“The criminal justice service report recommended referral to a Sacro programme aimed at* ***men who abuse their female partner****. “Meanwhile the victim, a* ***biological woman****, now identifies as non-gender so the court could not accede”…“It can only be used for* ***heterosexual relationships*** *where the male is the abuser, which does seem to be extremely strange****. If the abused partner identifies as non-female then the man’s relationship is no longer heterosexual****, is my understanding of the logic.”* | The Times  This newspaper article reports on a male convicted of domestic violence against his female partner who was unable to take part in a rehabilitation program for male offenders in *heterosexual* relationships because his partner had a non-binary gender identity. It seems that the organisation interpreted *heterosexual* as meaning an opposite gender identity couple (man gender identity and woman gender identity) rather than an opposite sex couple (male and female). This gendered interpretation seems to undermine the purpose of the program which is to ‘address domestic abuse by *men* towards a *female* partner or ex-partner and to reduce their reoffending and improve the lives of women, children and the men.’  <https://archive.md/j68MP> |
| **Unscientific representations of sex** |  |
| García-Acosta, J. M., San Juan-Valdivia, R. M., Fernández-Martínez, A. D., et al. (2020). Trans* Pregnancy and Lactation: A Literature Review from a Nursing Perspective. *International Journal of Environmental Research and Public Health, 17*(1), 44  *Lactation and* ***pregnancy*** *are viable processes that* ***do not depend on sex****. Even for the latter, it is only necessary to have an organ capable of gestation.* | This review of transgender pregnancy and lactation suggests that the capacity to experience the state of pregnancy is not dependent on the sex of the person.  <https://doi.org/10.3390/ijerph17010044> |
| Bamberger, E. T., & Farrow, A. (2021). Gendered and inclusive language in the preparation of manuscripts: policy statement for the Journal of Human Lactation. *Journal of Human Lactation, 37*(2), 227-229.  *Writers need to examine what assumptions they are expecting—consciously or not—the reader to make. As a simple example, the term* ***“the opposite sex”*** *can only be understood by making assumptions about* ***what sexes exist*** *and their relation to each other. Usage of this term would most likely result in the reader assuming that the writer was referring to a* ***dyadic model of sex****.* | This policy document from the journal of the International Lactation Consultant Association erroneously suggests that there are more than two sexes reflecting a Queer Theory-informed understanding.  [https://doi.org/10.1177/0890334421995103](https://doi.org/10.1177%2F0890334421995103) |
| The gender gap in cystic fibrosis *A comprehensive analysis in 1997 of more than 21,000 people with cystic fibrosis in the United States showed a median life expectancy of 25.3 years for* ***women*** *and 28.4 for* ***men****. The bacteria associated with lung decline and early death were also found to be present in* ***women*** *earlier than in* ***men****. (Nature recognizes that* ***sex and gender*** *are not the same, and* ***are neither fixed nor binary****.)* | Nature Magazine  This article on sex differences in cystic fibrosis describes the poorer outcomes that females with the disease experience and summarises the research exploring the phenomenon. However, while the whole article is based on the differences between the two sexes, it then includes an (incorrect) disclaimer stating that *sex is not fixed nor binary*. The title also incorrectly states that it is about the *gender gap in cystic fibrosis* when the article is solely about *sex* differences.  <https://www.nature.com/articles/d41586-020-02110-0> |
| **Technological encouragement for desexing language** | |
| *Original text*  ***Maternity leave*** *positively impacts maternal mental health and physical well-being*  *Microsoft Word Suggestions*  *Inclusiveness: A* ***gender-neutral*** *term here would be more inclusive*  *Parental leave*  *Family Leave*  *Childcare leave*  *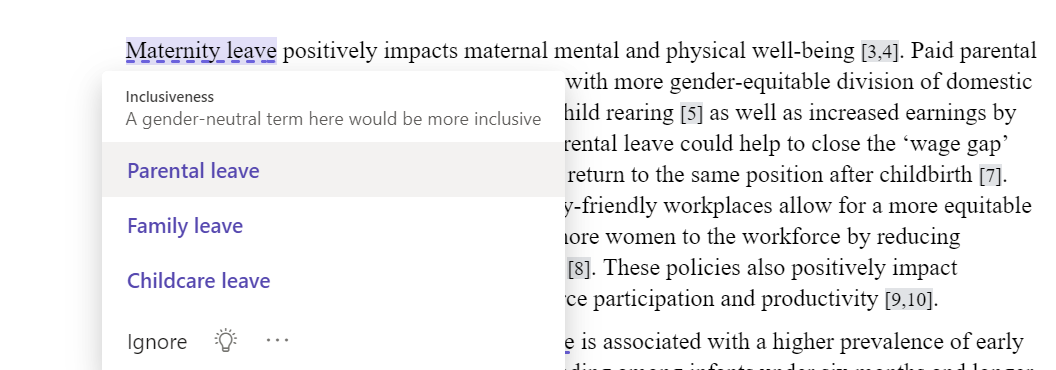* | Some versions of Microsoft Word provide suggestions for language changes. One of the authors of this paper was writing about maternity leave in the context of a middle-income country and was prompted (unasked) by Word that a *gender-neutral term* would be more inclusive than *maternity leave*. The terms proposed replacement terms were *parental leave*, *family leave*, and *childcare leave*. In this context, the use of *gender-neutral* was misleading as *maternity leave* was being used as a sexed term and not a gendered one. In addition, the proposed replacements are not synonyms for maternity leave and use of any of the proposed replacements would include those who should not be included and be misleading. |
| Some search engine optimisation (SEO) software that provides authors with direction on how to improve the ranking of pages in search engines is suggesting that articles dealing with pregnancy include the terms *pregnant person* and related desexed terms. |  |
