# Supplementary material for: Effective Communication About Pregnancy, Birth, Lactation, Breastfeeding and Newborn Care: The Importance of Sexed Language
Source: Front Glob Womens Health. 2022 Feb 7;3:818856. doi: 10.3389/fgwh.2022.818856 (PMC8864964; doi:10.3389/fgwh.2022.818856)
Supplement: Supplementary file 2 [file Table_2.docx]

**Supplementary Material 2- Examples of use of inclusivity statements and definitions to make it clear that those who have a gender identity meaning they do not identify as a woman or mother are included when sexed terminology is used.**

| **Document** | **Quotation** |
| --- | --- |
| Bartick, M., Hernández-Aguilar, M. T., Wight, N., Mitchell, K. B., Simon, L., Hanley, L., Meltzer-Brody, S., & Lawrence, R. M. (2021). ABM Clinical Protocol #35: Supporting breastfeeding during maternal or child hospitalization. *Breastfeeding Medicine, 16*(9), 664-674. <https://www.liebertpub.com/doi/10.1089/bfm.2021.29190.mba> | *The Academy of Breastfeeding Medicine recognizes that not all lactating individuals identify as women. Using gender-inclusive language, however, is not possible in all languages and all countries and for all readers. The position of the Academy of Breastfeeding Medicine is to interpret clinical protocols within the framework of inclusivity of all breastfeeding, chestfeeding, and human milk-feeding individuals.* |
| Australian Breastfeeding Association (2021). Breastfeeding: Relactation and Induced Lactation. Melbourne, Australian Breastfeeding Association.  <https://shop.breastfeeding.asn.au/products/relactation-and-induced-lactation/> | *Most people who relactate or induce lactation see themselves as women and as mothers, so these are the terms used in this booklet. No offence is intended to any person who uses other words to describe themselves. Readers who prefer non-sexed language may prefer the book Breastfeeding, chest feeding and human milk feeding: Supporting LGBTQIA+ families, produced by the Australian Breastfeeding Association with Rainbow Families NSW* |
| Becker, G. E., Ching, C., Zambrano, P., Burns, A., Cashin, J., & Mathisen, R. (2021). Evidence of violations of the International Code of Marketing of Breast-Milk Substitutes since the adoption by the World Health Assembly in 1981: a systematic scoping review protocol. *International Journal of Environmental Research and Public Health, 18*(18), 9523 <https://doi.org/10.3390/ijerph18189523> | *The terms ‘woman’ and ‘mother’ and ‘breastfeeding’ are used throughout our paper. Individual parents and families may use different words and we respect their preferred terminology.* |
| British Pregnancy Advisory Service. (2021). *Our Values. Our Vision. Our Ambitions.* <https://www.bpas.org/media/3550/bpas-advocacy-values-vision-ambitions.pdf> | *Our services are inclusive, and we will build specialist pathways that meet individual needs – particularly for those who do not identify as women in recognition that pregnancy may be especially challenging for those experiencing dysphoria. However we will continue to talk about women in our campaigning, advocacy and general client materials. This is partly in acknowledgement that this is how the majority of those using our services see themselves, and patients need to recognise themselves in health-related information in order for it to be effective. But we will also continue to use the word “women” over “people” so we can continue to campaign effectively for reproductive rights. Women’s reproductive healthcare and choices remain regulated and restricted in the way they are precisely because they are women’s issues, sadly still bound up with heavily gendered and judgmental approaches to female sexuality, ideals of motherhood and expectations of maternal sacrifice, and the need to control women’s bodies and choices. If we cannot clearly articulate that it is predominantly women, rather than people at large, who are affected by this we will find it much harder to dismantle a framework that today is still underpinned by sexism, and achieve a broader goal of ensuring that everyone, no matter how they identify, can access the care and support they need as swiftly and straightforwardly as possible.* |
| Emergency Nutrition Network, & Infant and Young Child Feeding in Emergencies Core Group. (2021). *Operational Guidance: Breastfeeding Counselling in Emergencies*. Oxford: Emergency Nutrition Network.  <https://www.ennonline.net/breastfeedingcounsellinginemergencies> | *The phrase “mothers and other caregivers” has been used in this document in acknowledgement that not all people who birth a child identify as a mother and, particularly in emergencies which are often characterised by high morbidity and mortality rates and increased mother-child separation, not all primary caregivers of infants and young children are mothers. What terminology is appropriate to use is culture and caregiver dependent. In line with the basic principles of counselling, counsellors should actively listen and reflect whatever language the person they are counselling is using, including how they want to be referred to. When unsure, it is better to ask than to make assumptions.* |
| Gribble, K. D. (2020). Writing expert reports for court in relation to breastfeeding infants and young children. *Journal of Human Lactation*, *37*(4), 683-688. <https://journals.sagepub.com/doi/abs/10.1177/0890334420975393> | *In this paper, ‘mother’ refers to the female biological parent of a child and ‘woman’ to a person of the female sex.* |
| Hare, H., & Womersley, K. (2021). Why were breastfeeding women denied the covid-19 vaccine? *BMJ Opinion*. <https://blogs.bmj.com/bmj/2021/01/05/why-were-breastfeeding-women-denied-the-covid-19-vaccine/> | *We have referred to ‘women’, ‘mothers’, and ‘breastfeeding’. Other parents and families use different words, and we respect their chosen terminology.* |
| National Institute for Health and Care Excellence, & Royal College of Obstetricians and Gynaecologists. (2021). *Postnatal Care: NICE Guideline*. London: NICE  <https://www.nice.org.uk/guidance/ng194>  National Institute for Health and Care Excellence (2021). *Caesarean Birth: NICE Guideline*. London: NICE  <https://www.nice.org.uk/guidance/ng192> | *The guideline uses the terms 'woman' or 'mother' throughout. These should be taken to include people who do not identify as women but are pregnant or have given birth.* |
| Nursing and Midwifery Council. (2019). *Standards of Proficiency for Midwives,* London: Nursing and Midwifery Council. <https://www.nmc.org.uk/globalassets/sitedocuments/standards/standards-of-proficiency-for-midwives.pdf> | *Woman: the words woman and women have been used throughout this document as this is the way that the majority of those who are pregnant and having a baby will identify. For the purpose of this document, this term includes girls. It also includes people whose gender identity does not correspond with their birth sex or who may have a non-binary identity*. |
| Scottish Government. (2021). Women’s Health Plan. <https://www.gov.scot/publications/womens-health-plan/pages/3/> | *This document will use the term 'women'/'woman' throughout. It is important to highlight that it is not only those who identify as women who require access to women's health and reproductive services. For example, some transgender men, non-binary people, and intersex people or people with variations in sex characteristics may also experience menstrual cycles, pregnancy, endometriosis and the menopause. The actions included within this Plan make clear that all healthcare services should be respectful and responsive to individual needs.* |
| US Preventive Services Task Force. (2019). Risk assessment, genetic counseling, and genetic testing for BRCA-related cancer: US Preventive Services Task Force recommendation statement. *JAMA, 322*(7), 652-665. <https://jamanetwork.com/journals/jama/fullarticle/2748515> | *While this recommendation applies to women, the net benefit estimates are driven by biological sex (ie, male/female) rather than gender identity. Persons should consider their sex at birth to determine which recommendation best applies to them.* |
| Williams, J., Plater, D., Brunacci, A., et al. (2019). *Abortion: A Review of South Australian Law and Practice*. South Australian Law Reform Institute. <https://law.adelaide.edu.au/system/files/media/documents/2019-12/Abortion%20Report%20281119.pdf> | *SALRI [South Australian Law Reform Institute] uses the term ‘woman’ to refer to someone needing an abortion. SALRI intends no disrespect by this and has adopted the approach of the South Australian Abortion Action Coalition [SAAAC], which noted to SALRI: People needing abortions are referred to in this submission as women and the vast majority of abortions are provided to people who identify as women. SAAAC acknowledges that other people who do not identify as women can need access to abortion. Trans-men, gender queer and others of diverse gender identities who do not necessarily identify as women, can and do get pregnant and require abortion care.* |
| Women and Birth. (2021). *Author information pack.*  <https://www.elsevier.com/wps/find/journaldescription.cws_home/707424?generatepdf=true> | *The authors recognise that individuals have diverse gender identities. Terms such as pregnant person, childbearing people and parent can be used to avoid gendering birth, and those who give birth, as feminine. However, because women are also marginalised and oppressed in most places around the world, we have continued to use the terms woman, mother or maternity. When we use these words, it is not meant to exclude those who give birth and do not identify as women.* |
| WRISK Project. (2021). Research and Engagement.  <https://wrisk.org/research-and-engagement/> | *The WRISK project is inclusive of trans, non-binary and intersex people. For this reason, the project team will always refer to individuals according to their self-determined gender. We tend to use the words ‘woman/women’ for convenience, as the great majority of people covered by the WRISK research self-identify as women. However, the WRISK project is inclusive of everyone who has been pregnant in the past five years; regardless of their gender identification. This includes trans men and non-binary individuals. If you have any questions about this please contact the research team.* |
